# Supplementary material for: Genetic risk for schizophrenia and developmental delay is associated with shape and microstructure of midline white-matter structures
Source: Transl Psychiatry. 2019 Feb 25;9:102. doi: 10.1038/s41398-019-0440-7 (PMC6389944; doi:10.1038/s41398-019-0440-7)
Supplement: Supplementary file 1 — Supplemental Material [file 41398_2019_440_MOESM1_ESM.pdf]

"Genetic risk for schizophrenia and developmental delay is associated with shape and microstructure of midline white matter structures"

Supplementary material

1. Demographic and neuropsychiatric information of CNV patients

Table S1 lists additional information for CNVs carriers included in the present study, IQ scores obtained using the Wechsler Adult Intelligence Scale (WAIS-III) (mean±s.d. FSIQ: 93±17.3; VIQ: 91.0±20.3; PIQ; 96.7±14.6). Of the CNV carriers, we identified 17/21 (81%) who satisfied criteria for at least one psychiatric diagnosis according to the Psychiatric Assessment Schedule for Adults with Developmental Disability (PAS-ADD)[1]: 9/21 (43%) had diagnoses for anxiety disorders and 6/21 (29%) for neurodevelopmental disorders (including autism spectrum disorder, social communication disorder, attention deficit / hyperactivity disorder and intellectual disability). Only 4/21 (19%) did not meet criteria for psychiatric diagnosis. The number of diagnoses amongst those with diagnoses ranged from 1 to 7 (median: 2).

[1] Moss SC, Ibbotson B, Prosser H, Goldberg DP, Patel P & Simpson N (1997) Validity of the PAS-ADD for detecting psychiatric symptoms in adults with learning disability. Social Psychiatry and Psychiatric Epidemiology 32 344–354.

Table S1. Demographic details of each CNV participant, psychiatric diagnoses and IQ scores.

CNV (hg19)	Age (years)		Gender		N	Median number of diagnoses	FSIQ		VIQ		PIQ	
	Mean	s.d.	M	F			Mean	s.d.	Mean	s.d.	Mean	s.d.
**All CNVs**	**37.4**	**11.7**	**14**	**7**	**21**	**2**	**93**	**17.3**	**91.0**	**20.3**	**96.7**	**14.6**
15q11.2 BP1-2 deletion (chr15:22,805,313-23,094,530)	48.4	2.3	1	1	2	1	107	7.1	103	7.1	104.5	2.1
15q13.3 BP4-5 deletion (chr15:31,080,645-32,462,776)	30.0	4.5	2	0	2	4.5	65	16.3	63.5	12.0	71.5	20.5
15q13.3 BP4-5 duplication (chr15:31,080,645-32,462,776)	41.7	-	1	0	1	3	119	-	116	-	118	-
16p11.2 distal duplication (chr16:28,823,196-29,046,783)	40.3	-	0	1	1	1	95	-	94	-	97	-
16p11.2 deletion chr16:29,650,840-30,200,773	43.0	-	1	0	1	3	83	-	72	-	100	-
17q12 duplication (chr17:34,815,904-36,217,432)	47.1	-	0	1	1	2	83	-	86	-	90	-
1q21.1 deletion (chr1:146,527,987-147,394,444)	35.0	15.0	4	0	4	2	85	17.0	78.25	23.5	96.25	9.4
1q21.1 duplication (chr1:146,527,987-147,394,444)	39.5	-	0	1	1	5	104	-	88	-	121	-
22q11.2 deletion (chr22:19,037,332-21,466,726)	31.2	17.0	2	2	4	2.5	92	19.3	91	22.9	93	14.1
22q11.2 duplication (chr22:19,037,332-21,466,726)	44.9	4.8	1	1	2	1	113	9.9	120	1.4	103.5	20.5
3q29 deletion (chr3:195,720,167-197,354,826)	19.9	-	1	0	1	1	89	-	98	-	86	-
NRXN1 deletion (chr2:50145643-51259674)	43.6	-	1	0	1	1	97	-	99	-	94	-
**Control**	**39.6**	**11.3**	**6**	**9**	**15**	-	-	-	-	-	-	-

2. Head motion

Head motion during the diffusion MRI scans was estimated from the average norm of the head (relative to the first diffusion weighted acquisition) across the scan. The relationship between head motion and penetrance scores are shown in Figure S1. Spearmans rho correlation coefficient is significant for both penetrance scores. Head motion was included as a covariate in all statistical analysis performed.

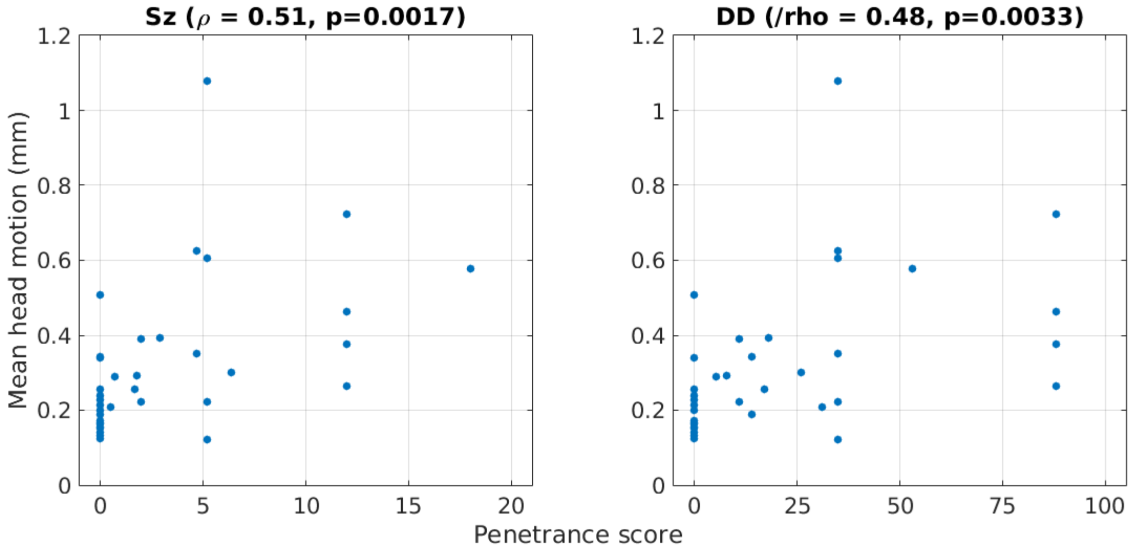

Figure S1. Relationships between mean head motion recorded during diffusion weighted acquisition and penetrance scores for Sz (left) and DD (right).

3. Leave-one-out analysis

To assess the contribution of each individual CNV to the results presented in the main text, we preform additional statistical analysis leaving each CNV group out and examine how the results of the statistical analysis changes. The analyses were repeated for the shape descriptor describing cingulum curvature, FA and ICVF. Multiple comparisons were corrected using permutation tests as described in the main text. The change in effect size was estimate by the difference in t-value normalised to the square root of the number participants removed.

The corrected p-values for each CNV omission are shown in Table S2. Estimated changes in effect size due to each CNV omission are shown in Figure S2. Results show that for all comparisons except for association of P_{Sz} with FA in the left cingulum bundle, removal of the 22q11.2 deletion patients resulted in the observed association becoming non-significant. For the P_{Sz}-FA association in the left cingulum bundle, this is rendered non-significant by removal of patients with 3q29 and 1q21.1 deletions.

This indicates the observed effect is influenced by the presence of 22q11.2 deletion, suggesting that other CNVs (which typically have lower penetrance for both Sz and DD) are less associated with the brain changes observed. However, it is difficult to make firm conclusions about these results, since the 22q11.2 deletions constitute a large proportion of the CNV sample, the loss of effect could simply be due to loss of statistical power.

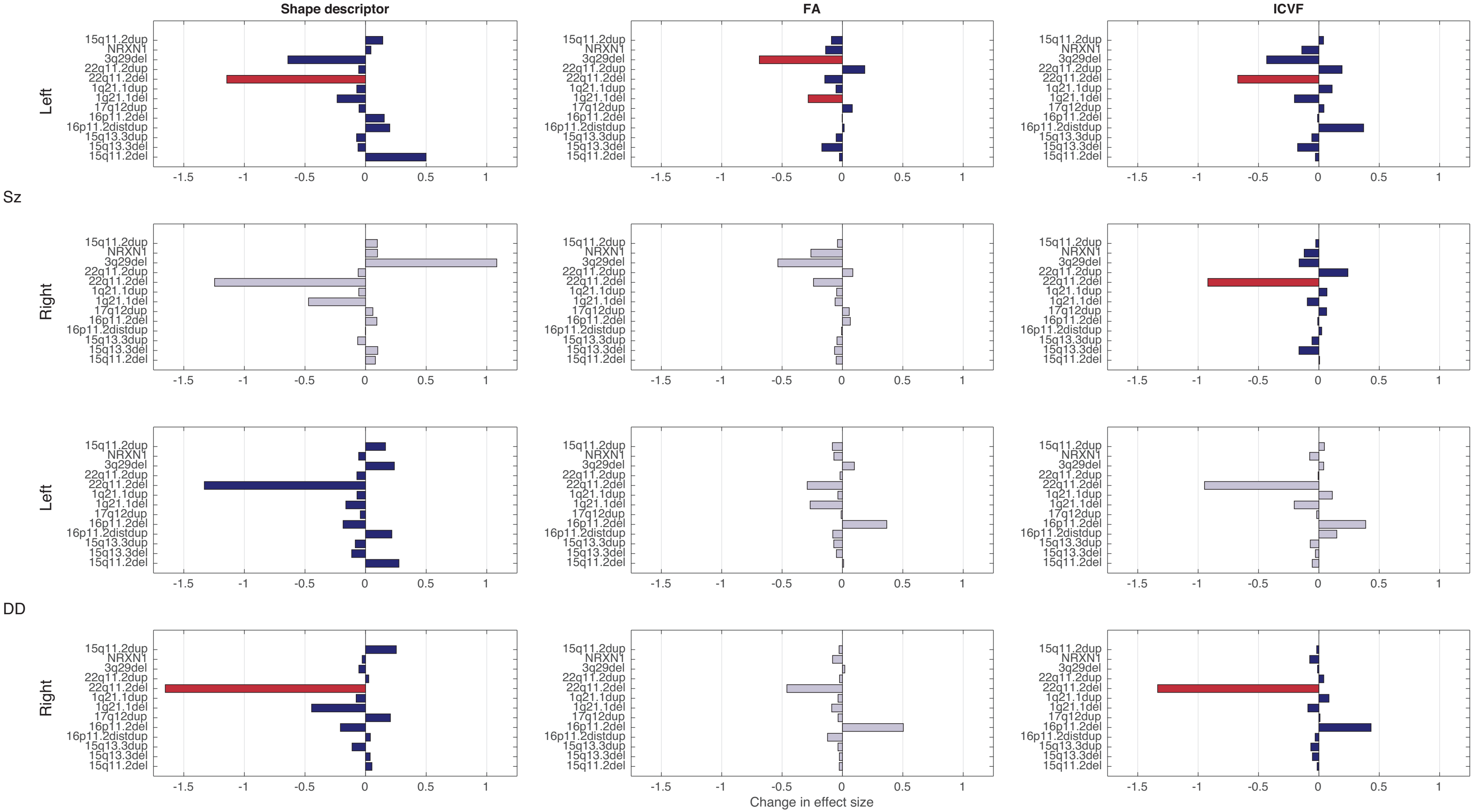

Figure S2. Estimated change in effect sizes due to each CNV omission from the main analysis. Red bars indicate where association is longer significant after CNV omission. Grey bars indicate where association was not significant prior to omission.

Table S2. Corrected p-values for each analysis performed with each CNV omission. Analysis focused on left and right cingulum for the measures previously reported significant in the main text (curvature, FA and ICVF) Red cells indicate where p-values are no longer significant after CNV omission. Grey cells indicate where results were not significant in the main analysis.

CNV omitted			15q11.2 del	15q13.3 del	15q13.3 dup	16p11.2 dist dup	16p11.2 del	17q12 dup	1q21.1 del	1q21.1 dup	22q11.2 del	22q11.2 dup	3q29 del	NRXN1 del	15q11.2 dup	None (original)
Curvature	Sz	Left	0.0000	0.0003	0.0003	0.0001	0.0001	0.0003	0.0009	0.0003	0.0675	0.0003	0.0013	0.0002	0.0002	0.0061
		Right	0.0009	0.0008	0.0013	0.0011	0.0009	0.0010	0.0130	0.0013	0.2770	0.0015	0.0001	0.0009	0.0009	0.0512
	DD	Left	0.0001	0.0005	0.0004	0.0002	0.0005	0.0004	0.0009	0.0004	0.1734	0.0004	0.0002	0.0004	0.0002	0.0086
		Right	0.0000	0.0000	0.0000	0.0000	0.0000	0.0000	0.0001	0.0000	0.0295	0.0000	0.0000	0.0000	0.0000	0.0004
FA	Sz	Left	0.0146	0.0282	0.0198	0.0172	0.0150	0.0142	0.0666	0.0144	0.0384	0.0066	0.0822	0.0240	0.0178	0.0172
		Right	0.1270	0.1400	0.1244	0.1096	0.1000	0.1072	0.1410	0.1126	0.2571	0.0870	0.2821	0.1766	0.1198	0.1050
	DD	Left	0.1290	0.1568	0.1652	0.1530	0.0696	0.1464	0.3195	0.1366	0.3605	0.1472	0.1304	0.1564	0.1550	0.1288
		Right	0.5175	0.5117	0.5299	0.5489	0.2731	0.5251	0.5837	0.5009	0.8872	0.5227	0.4899	0.5315	0.5025	0.4857
ICVF	Sz	Left	0.0164	0.0306	0.0188	0.0078	0.0126	0.0156	0.0394	0.0132	0.2188	0.0068	0.0156	0.0214	0.0124	0.0124
		Right	0.0254	0.0440	0.0272	0.0288	0.0238	0.0220	0.0386	0.0246	0.5093	0.0098	0.0250	0.0322	0.0204	0.0210
	DD	Left	0.0780	0.0690	0.0720	0.0490	0.0230	0.0690	0.1390	0.0530	0.7350	0.0530	0.0580	0.0720	0.0540	0.0630
		Right	0.0480	0.0550	0.0520	0.0510	0.0140	0.0440	0.0720	0.0400	0.9230	0.0350	0.0460	0.0500	0.0410	0.0390

4. Results of statistical tests

The following pages present tables summarising the results of all statistical comparisons performed.

SUMMARY OF MAIN RESULTS

Significant pos effect	Significant neg effect
Marginal pos effect	Marginal neg effect

			Sz t	p	p_corr	DD t	p	p_corr
**WM MORPHOLOGY**								
Significant effect of P_Sz and P_DD on dorsal cingulum curvature	Shape	CingL	4.195	0.000	0.026	4.060	0.000	0.035
This is stronger for Sz in the left cingulum, but stronger for DD in the right cingulum		Cing R	-3.601	0.001	0.111	-5.604	0.000	0.001
Effect strong but not surviving correction for Sz / right cingulum								
No effects surviving correction in any other tracts								
Signifcant negative correlation between P_Sz and volume of right uncinate fasiculus and corticospinal tracts					0.038			
**WM MICROSTRUCTURE**								
Significant negative effect of P_Sz on FA in left cingulum	CingL	FA	-3.290	0.002	0.017	-2.316	0.027	0.131
		MD	1.448	0.161	0.394	1.641	0.111	0.316
		RD	0.620	0.545	0.793	1.232	0.227	0.506
Significant neg effect of P_Sz on ICVF in left cingulum	CingL	ICVF	-3.258	0.003	0.019	-2.644	0.013	0.064
Marginal corresponding effects of P_DD in left cingulum		ISOF	-2.551	0.016	0.072	-1.509	0.141	0.370
Sig neg effect of P_DD on ICVF in right cingulum		ODI	1.905	0.066	0.342	1.403	0.171	0.577
N o other sig effects in L Cing								
Significant neg effect of P_Sz on ICVF in right cingulum	CingR	ICVF	-3.054	0.005	0.029	-2.814	0.008	0.047
Also significant neg effect of P_DD on R cingulum volume (marginal effect of P_Sz)	CingR	Volume	-2.406	0.022	0.146	-3.698	0.001	0.011
Sig pos effect on ODI in Left and right infoerior frontoocipital fasiculus	'IFOL'	ODI	3.757	0.001	0.007	2.724	0.010	0.089
	'IFOR'	ODI	4.034	0.000	0.004	2.229	0.033	0.210
Sig pos effect of both P_Sz and P_DD on ODI in left Uncinate fasiculus	UNCR	ODI	4.054	0.000	0.004	3.593	0.001	0.014
**GM MORPHOLOGY**								
No significant effects except for marginal effects on curvature of the left medialorbitofrontal cortex			3.671	0.001	0.080			
**GROSS BRAIN VOLUMETRY**								
No effects								
**PRINCIPLE COMPONENTS**								
PC8 showed significant negative correlation with both P_DD and marginal with P_Sz		PC8	-2.560	0.016	0.055	-3.033	0.005	0.027
This is weighted toward white matter volume features.								
Post hot test of CC body:splenium ratio confirms effect		Ccratio	-2.180	0.037			-2.979	0.006
This seems to make sense in conjunction with the cingulum morphology findings. The ratio of these segments of the ocrpus calosum would coincide thw th shape of the cingulum which sits on top of it.								
Also a marginal effect of P_Sz on PC1								
PC1 is weighted towards gross whole-brain volume measurments		PC1	-2.657	0.012	0.055	-1.746	0.091	0.818

ALL MICROSTRUCTURAL FINDINGS WITH BINARY CNV MODEL
UNCORRECTED P

	'FA'	'MD'	'RD'	'AD'	'R1'	'T1'	'ICVF'	'ISOF'	'ODI'	Volume
'ARCL'	0.307	0.304	0.536	0.115	0.321	0.294	0.925	0.935	0.802	0.126
'ARCR'	0.731	0.929	0.983	0.944	0.328	0.291	0.466	0.584	0.983	0.144
'CCbody'	0.352	0.222	0.243	0.214	0.211	0.214	0.802	0.538	0.619	0.071
'CCgenu'	0.605	0.797	0.860	0.665	0.214	0.223	0.578	0.442	0.371	0.230
'CCsplen'	0.228	0.914	0.877	0.613	0.226	0.221	0.739	0.621	0.351	0.952
'Cing_L'	0.176	0.355	0.323	0.270	0.245	0.237	0.613	0.596	0.320	0.484
'Cing_R'	0.571	0.499	0.577	0.438	0.143	0.140	0.416	0.935	0.897	0.100
'FORNIX_left'	0.741	0.519	0.500	0.735	0.741	0.525	0.168	0.601	0.329	0.214
'FORNIX_right'	0.885	0.274	0.215	0.852	0.247	0.190	0.921	0.292	0.245	0.214
'IFOL'	0.137	0.205	0.317	0.161	0.229	0.208	0.669	0.890	0.359	0.425
'IFOR'	0.923	0.472	0.561	0.420	0.249	0.213	0.914	0.528	0.964	0.083
'PHCL'	0.849	0.753	0.897	0.579	0.244	0.234	0.878	0.875	0.843	0.361
'PHCR'	0.753	0.652	0.550	0.721	0.411	0.310	0.237	0.171	0.097	0.746
'UNCL'	0.122	0.104	0.224	0.041	0.780	0.717	0.901	0.297	0.102	0.657
'UNCR'	0.188	0.380	0.498	0.267	0.975	0.919	0.746	0.320	0.075	0.045
'corticoSpinalL'	0.323	0.315	0.376	0.735	0.505	0.380	0.573	0.629	0.525	0.209
'corticoSpinalR	0.032	0.420	0.888	0.102	0.460	0.349	0.446	0.244	0.031	0.014
'ilfLeft'	0.130	0.129	0.218	0.086	0.270	0.256	0.640	0.757	0.279	0.223
'ilfRight'	0.816	0.632	0.519	0.961	0.204	0.189	0.687	0.650	0.756	0.360

T

	'FA'	'MD'	'RD'	'AD'	'R1'	'T1'	'ICVF'	'ISOF'	'ODI'	Volume
'ARCL'	-1.056	1.036	0.623	1.637	-1.008	1.069	-0.096	0.082	0.253	1.572
'ARCR'	0.346	0.165	0.071	-0.015	-0.995	1.074	0.739	0.554	0.022	1.500
'CCbody'	-0.780	1.040	0.998	1.073	-1.278	1.269	-0.253	0.623	-0.502	-1.870
'CCgenu'	-0.595	0.378	0.274	0.585	-1.269	1.244	0.562	0.779	0.907	-1.225
'CCsplen'	-1.285	0.146	-0.112	0.552	-1.237	1.249	-0.337	-0.500	0.946	0.061
'Cing_L'	-1.291	0.804	0.897	0.981	-1.186	1.207	-0.511	0.535	1.010	-0.708
'Cing_R'	-0.418	0.494	0.389	0.597	-1.503	1.517	-0.824	0.082	-0.130	-1.697
'FORNIX_left'	-0.201	-0.329	-0.326	-0.120	-0.333	0.643	1.412	0.529	0.991	1.270
'FORNIX_right'	0.409	-1.021	-1.079	-0.335	-1.181	1.342	-0.100	-1.072	1.184	1.270
'IFOL'	-1.417	1.261	0.982	1.381	-1.228	1.288	-0.432	-0.139	0.930	0.809
'IFOR'	-0.046	0.677	0.531	0.758	-1.175	1.273	0.109	0.639	0.046	1.792
'PHCL'	-0.186	0.214	0.077	0.455	-1.189	1.213	-0.155	-0.159	0.200	-0.928
'PHCR'	-0.212	0.496	0.679	0.343	-0.833	1.033	1.207	1.402	1.713	0.327
'UNCL'	-1.459	1.531	1.106	1.975	0.282	-0.365	0.125	1.060	1.683	-0.449
'UNCR'	-1.306	0.752	0.515	1.016	0.031	-0.103	0.327	1.011	1.842	-2.093
'corticoSpinalL'	0.947	0.988	0.828	0.395	-0.675	0.891	0.570	0.488	-0.643	-1.284
'corticoSpinalR	-2.219	1.038	0.364	1.752	-0.749	0.951	0.772	1.188	2.258	-2.605
'ilfLeft'	-1.540	1.516	1.203	1.748	-1.124	1.158	-0.472	-0.312	1.101	-1.245
'ilfRight'	0.079	0.571	0.687	0.219	-1.298	1.345	0.407	0.458	0.313	-0.930

MICROSTRUCT Sz pen

ALL MICROSTRUCTURAL FINDINGS ASSOCIATED WITH PEN_SZ

P

	'FA'	'MD'	'RD'	'AD'	'R1'	'T1'	'ICVF'	'ISOF'	'ODI'	Volume
'ARCL'	0.023	0.233	0.050	0.423	0.576	0.664	0.600	0.820	0.007	0.333
'ARCR'	0.303	0.471	0.312	0.469	0.510	0.612	0.525	0.523	0.216	0.539
'CCbody'	0.858	0.758	0.974	0.457	0.924	0.975	0.549	0.330	0.480	0.155
'CCgenu'	0.220	0.519	0.978	0.050	0.797	0.974	0.484	0.174	0.028	0.268
'CCsplen'	0.377	0.720	0.755	0.211	0.897	0.977	0.525	0.301	0.194	0.810
'Cing_L'	0.002	0.161	0.050	0.270	0.293	0.615	0.003	0.016	0.066	0.223
'Cing_R'	0.021	0.241	0.157	0.492	0.223	0.384	0.005	0.025	0.166	0.022
'FORNIX_left'	0.040	0.225	0.007	0.913	0.368	0.723	0.833	0.905	0.626	0.402
'FORNIX_right'	0.343	0.456	0.089	0.836	0.242	0.511	0.175	0.297	0.831	0.402
'IFOL'	0.018	0.721	0.139	0.005	0.616	0.725	0.620	0.399	0.001	0.668
'IFOR'	0.018	0.490	0.167	0.002	0.431	0.458	0.644	0.518	0.000	0.639
'PHCL'	0.354	0.504	0.866	0.710	0.739	0.744	0.932	0.865	0.226	0.560
'PHCR'	0.367	0.239	0.355	0.307	0.678	0.720	0.735	0.575	0.045	0.254
'UNCL'	0.171	0.637	0.281	0.357	0.287	0.281	0.732	0.774	0.024	0.462
'UNCR'	0.009	0.449	0.148	0.037	0.375	0.367	0.427	0.618	0.000	0.007
'corticoSpinalL'	0.785	0.308	0.659	0.464	0.746	0.829	0.624	0.985	0.520	0.175
'corticoSpinalR	0.151	0.279	0.089	0.892	0.678	0.760	0.477	0.743	0.628	0.003
'ilfLeft'	0.316	0.759	0.444	0.488	0.932	0.965	0.884	0.992	0.114	0.214
'ilfRight'	0.374	0.485	0.532	0.850	0.549	0.687	0.698	0.939	0.463	0.491

T

	'FA'	'MD'	'RD'	'AD'	'R1'	'T1'	'ICVF'	'ISOF'	'ODI'	Volume
'ARCL'	-2.359	1.198	2.041	-0.811	-0.565	0.439	-0.530	0.229	2.887	0.984
'ARCR'	-1.031	0.711	1.027	-0.733	-0.667	0.512	-0.643	-0.646	1.263	-0.621
'CCbody'	0.152	0.342	-0.033	0.753	-0.096	-0.032	-0.605	-0.989	-0.715	-1.459
'CCgenu'	-1.223	-0.660	-0.028	-2.035	-0.260	0.033	-0.708	-1.391	2.302	-1.129
'CCsplen'	-0.874	-0.361	0.315	-1.276	-0.130	-0.029	-0.642	-1.053	1.329	0.243
'Cing_L'	-3.290	1.448	2.038	-1.124	-1.069	0.508	-3.258	-2.551	1.905	-1.243
'Cing_R'	-2.472	1.230	1.449	-0.695	-1.245	0.884	-3.054	-2.355	1.419	-2.406
'FORNIX_left'	-2.167	1.259	2.884	-0.110	-0.913	0.358	-0.212	0.120	0.493	0.850
'FORNIX_right'	-1.263	0.735	1.754	-0.209	-1.193	0.665	-1.388	-1.061	0.215	0.850
'IFOL'	-2.500	0.358	1.518	-3.026	-0.507	0.356	-0.501	-0.855	3.757	0.432
'IFOR'	-2.466	0.695	1.415	-3.443	-0.798	0.752	-0.467	-0.653	4.034	-0.473
'PHCL'	-0.927	0.682	0.171	0.375	0.337	-0.329	-0.086	-0.172	1.235	-0.589
'PHCR'	-0.926	1.178	0.939	1.039	0.420	-0.362	0.341	0.567	2.089	-1.163
'UNCL'	-1.418	0.493	1.098	-0.935	-1.084	1.098	-0.345	-0.289	2.372	-0.745
'UNCR'	-2.748	0.781	1.482	-2.183	-0.900	0.915	-0.804	-0.503	4.054	-2.878
'corticoSpinalL'	0.281	1.025	0.445	0.742	-0.327	0.217	-0.495	-0.020	-0.651	-1.387
'corticoSpinalR	-1.454	1.081	1.755	-0.136	-0.419	0.309	-0.720	-0.331	0.489	-3.163
'ilfLeft'	-1.005	0.310	0.776	-0.701	-0.086	-0.045	-0.148	-0.010	1.626	-1.267
'ilfRight'	-0.875	0.688	0.632	0.190	-0.607	0.408	-0.392	-0.078	0.744	-0.697

P_CORR

	'FA'	'MD'	'RD'	'AD'	'R1'	'T1'	'ICVF'	'ISOF'	'ODI'	Volume
'ARCL'	0.117	0.493	0.551	0.999	0.660	0.667	0.826	0.887	0.060	0.757
'ARCR'	0.680	0.724	0.981	1.000	0.610	0.635	0.787	0.795	0.652	0.911
'CCbody'	0.948	0.871	1.000	0.999	0.839	0.859	0.799	0.637	0.880	0.560
'CCgenu'	0.577	0.796	1.000	0.467	0.784	0.838	0.760	0.435	0.179	0.720
'CCsplen'	0.752	0.890	1.000	0.939	0.828	0.858	0.787	0.602	0.619	0.965
'Cing_L'	0.017	0.394	0.270	0.975	0.424	0.636	0.019	0.072	0.342	0.670
'Cing_R'	0.113	0.503	0.485	1.000	0.357	0.464	0.029	0.103	0.574	0.146
'FORNIX_left'	0.181	0.481	0.284	1.000	0.498	0.698	0.912	0.914	0.932	0.816
'FORNIX_right'	0.717	0.714	0.587	1.000	0.374	0.564	0.440	0.598	0.974	0.816
'IFOL'	0.099	0.856	0.785	0.076	0.687	0.699	0.836	0.702	0.007	0.935
'IFOR'	0.101	0.734	0.668	0.030	0.550	0.522	0.847	0.793	0.004	0.940
'PHCL'	0.729	0.744	1.000	1.000	0.809	0.808	0.934	0.937	0.670	0.917
'PHCR'	0.743	0.500	0.930	0.988	0.778	0.795	0.873	0.787	0.262	0.703
'UNCL'	0.503	0.819	0.980	0.995	0.418	0.376	0.881	0.912	0.160	0.878
'UNCR'	0.056	0.709	0.696	0.378	0.504	0.449	0.722	0.850	0.004	0.057
'corticoSpinalL'	0.932	0.585	1.000	1.000	0.758	0.759	0.838	0.941	0.899	0.597
'corticoSpinalR	0.465	0.551	0.752	1.000	0.720	0.722	0.755	0.904	0.933	0.030
'ilfLeft'	0.689	0.871	1.000	1.000	0.842	0.865	0.925	0.940	0.469	0.657
'ilfRight'	0.749	0.731	0.997	1.000	0.640	0.678	0.868	0.952	0.867	0.893

MICROSTRUCT DD pen

ALL MICROSTRUCTURAL FINDINGS ASSOCIATED WITH PEN_DD

P

	'FA'	'MD'	'RD'	'AD'	'R1'	'T1'	'ICVF'	'ISOF'	'ODI'	Volume
'ARCL'	0.039	0.070	0.082	0.423	0.371	0.366	0.697	0.501	0.061	0.323
'ARCR'	0.441	0.357	0.337	0.469	0.292	0.299	0.726	0.957	0.232	0.632
'CCbody'	0.560	0.583	0.983	0.457	0.415	0.388	0.958	0.964	0.098	0.028
'CCgenu'	0.525	0.590	0.770	0.050	0.333	0.352	0.931	0.506	0.223	0.247
'CCsplen'	0.626	0.797	0.869	0.211	0.409	0.385	0.490	0.571	0.716	0.948
'Cing_L'	0.027	0.111	0.030	0.270	0.100	0.177	0.013	0.141	0.171	0.062
'Cing_R'	0.166	0.191	0.068	0.492	0.047	0.067	0.008	0.107	0.905	0.001
'FORNIX_left'	0.125	0.054	0.032	0.913	0.141	0.233	0.684	0.300	0.734	0.351
'FORNIX_right'	0.424	0.539	0.091	0.836	0.067	0.121	0.490	0.624	0.537	0.351
'IFOL'	0.043	0.508	0.154	0.005	0.218	0.230	0.893	0.769	0.010	0.870
'IFOR'	0.073	0.361	0.114	0.002	0.207	0.184	0.946	0.930	0.033	0.451
'PHCL'	0.994	0.556	0.535	0.710	0.790	0.607	0.798	0.784	0.132	0.268
'PHCR'	0.665	0.283	0.249	0.307	0.742	0.559	0.436	0.303	0.068	0.361
'UNCL'	0.303	0.395	0.334	0.357	0.636	0.635	0.898	0.789	0.068	0.381
'UNCR'	0.037	0.321	0.123	0.037	0.691	0.653	0.553	0.920	0.001	0.001
'corticoSpinalL'	0.517	0.136	0.580	0.464	0.488	0.425	0.907	0.690	0.311	0.458
'corticoSpinalR	0.138	0.114	0.140	0.892	0.341	0.301	0.962	0.463	0.490	0.064
'ilfLeft'	0.295	0.491	0.527	0.488	0.461	0.437	0.984	0.833	0.140	0.138
'ilfRight'	0.506	0.455	0.423	0.850	0.234	0.251	0.922	0.758	0.739	0.474

T

	'FA'	'MD'	'RD'	'AD'	'R1'	'T1'	'ICVF'	'ISOF'	'ODI'	Volume
'ARCL'	-2.156	1.875	1.797	-0.811	-0.908	0.918	-0.393	0.680	1.942	1.005
'ARCR'	-0.781	0.935	0.976	-0.733	-1.073	1.056	-0.354	0.054	1.218	-0.484
'CCbody'	0.589	0.555	0.021	0.753	-0.827	0.875	0.053	-0.045	-1.706	-2.309
'CCgenu'	-0.643	-0.544	0.294	-2.035	-0.983	0.946	-0.088	-0.672	1.244	-1.180
'CCsplen'	-0.492	0.260	0.166	-1.276	-0.838	0.881	-0.699	-0.573	0.367	0.066
'Cing_L'	-2.316	1.641	2.272	-1.124	-1.699	1.382	-2.644	-1.509	1.403	-1.934
'Cing_R'	-1.419	1.339	1.891	-0.695	-2.076	1.901	-2.814	-1.660	0.120	-3.698
'FORNIX_left'	-1.575	2.009	2.241	-0.110	-1.510	1.216	0.411	1.053	0.342	0.948
'FORNIX_right'	-0.811	0.622	1.745	-0.209	-1.898	1.596	-0.698	-0.496	0.624	0.948
'IFOL'	-2.105	0.670	1.462	-3.026	-1.258	1.224	-0.136	-0.297	2.724	-0.165
'IFOR'	-1.857	0.927	1.627	-3.443	-1.288	1.361	-0.068	0.089	2.229	-0.763
'PHCL'	-0.008	0.596	0.627	0.375	-0.269	0.520	0.258	0.276	1.546	-1.129
'PHCR'	-0.438	1.094	1.174	1.039	-0.332	0.591	0.788	1.046	1.888	-0.927
'UNCL'	-1.047	0.863	0.981	-0.935	-0.478	0.480	-0.129	0.269	1.892	-0.888
'UNCR'	-2.181	1.008	1.586	-2.183	-0.402	0.454	-0.600	-0.101	3.593	-3.617
'corticoSpinalL'	0.655	1.532	0.559	0.742	-0.702	0.808	-0.118	0.403	-1.030	-0.751
'corticoSpinalR	-1.523	1.630	1.516	-0.136	-0.967	1.052	-0.048	0.742	0.699	-1.925
'ilfLeft'	-1.066	0.698	0.639	-0.701	-0.747	0.788	-0.020	0.213	1.514	-1.522
'ilfRight'	-0.673	0.756	0.811	0.190	-1.214	1.172	-0.099	0.311	0.337	-0.725

P_CORR

	'FA'	'MD'	'RD'	'AD'	'R1'	'T1'	'ICVF'	'ISOF'	'ODI'	vol
'ARCL'	0.169	0.461	0.394	1.000	0.493	0.436	0.868	0.739	0.317	0.751
'ARCR'	0.787	0.976	0.975	1.000	0.419	0.379	0.878	0.928	0.672	0.940
'CCbody'	0.859	1.000	1.000	0.449	0.531	0.454	0.938	0.945	0.405	0.171
'CCgenu'	0.840	1.000	1.000	0.950	0.460	0.425	0.936	0.779	0.658	0.697
'CCsplen'	0.888	1.000	1.000	1.000	0.527	0.453	0.772	0.821	0.957	0.982
'Cing_L'	0.131	0.618	0.396	1.000	0.205	0.265	0.064	0.370	0.577	0.315
'Cing_R'	0.484	0.819	0.796	1.000	0.128	0.144	0.047	0.305	0.985	0.009
'FORNIX_left'	0.409	0.382	0.087	1.000	0.258	0.322	0.851	0.596	0.961	0.779
'FORNIX_right'	0.775	0.999	0.579	1.000	0.161	0.205	0.773	0.853	0.909	0.779
'IFOL'	0.186	0.999	0.748	0.415	0.349	0.319	0.928	0.909	0.089	0.980
'IFOR'	0.278	0.978	0.818	0.546	0.336	0.269	0.939	0.923	0.210	0.868
'PHCL'	0.969	0.999	1.000	0.918	0.777	0.614	0.895	0.876	0.497	0.718
'PHCR'	0.902	0.934	0.989	0.616	0.752	0.582	0.708	0.598	0.337	0.810
'UNCL'	0.666	0.987	0.959	1.000	0.693	0.633	0.929	0.878	0.336	0.824
'UNCR'	0.163	0.961	0.772	0.835	0.723	0.645	0.811	0.952	0.014	0.011
'corticoSpinalL'	0.838	0.694	1.000	0.819	0.592	0.485	0.931	0.837	0.757	0.871
'corticoSpinalR	0.432	0.625	0.577	1.000	0.466	0.381	0.943	0.715	0.886	0.322
'ilfLeft'	0.658	0.998	0.998	1.000	0.570	0.493	0.948	0.891	0.517	0.521
'ilfRight'	0.830	0.995	1.000	1.000	0.367	0.341	0.935	0.866	0.961	0.881

TRACTSHAPE

ALL TRACT SHAPE FINDINGS

Sz pen COMPONENT	T	P	P_CORR	DD pen COMPONENT	T	P	P_CORR	BIN T	P
'ARCL1'	0.392	0.698	1.000	'ARCL1'	0.505	0.617	1.000	0.754	0.316
'ARCL2'	-1.021	0.315	1.000	'ARCL2'	-1.824	0.078	0.999	0.589	-0.547
'ARCL3'	-0.206	0.838	1.000	'ARCL3'	-0.726	0.473	1.000	0.224	-1.241
'ARCL4'	0.196	0.846	1.000	'ARCL4'	1.283	0.209	1.000	0.889	0.140
'ARCL5'	0.654	0.518	1.000	'ARCL5'	0.151	0.881	1.000	0.584	-0.553
'ARCL6'	2.341	0.026	0.895	'ARCL6'	2.773	0.009	0.581	0.181	1.369
'ARCL7'	-1.533	0.135	1.000	'ARCL7'	-1.067	0.294	1.000	0.782	0.279
'ARCL8'	-0.158	0.875	1.000	'ARCL8'	0.726	0.473	1.000	0.731	0.346
'ARCL9'	0.375	0.710	1.000	'ARCL9'	0.815	0.421	1.000	0.105	-1.671
'ARCL10'	1.690	0.101	1.000	'ARCL10'	1.458	0.155	1.000	0.090	1.753
'ARCL11'	0.083	0.935	1.000	'ARCL11'	0.107	0.916	1.000	0.823	-0.225
'ARCR1'	1.258	0.218	1.000	'ARCR1'	0.978	0.336	1.000	0.591	0.543
'ARCR2'	-0.845	0.405	1.000	'ARCR2'	-1.106	0.277	1.000	0.056	-1.988
'ARCR3'	0.413	0.682	1.000	'ARCR3'	1.013	0.319	1.000	0.852	0.188
'ARCR4'	0.467	0.644	1.000	'ARCR4'	1.587	0.123	1.000	0.813	0.239
'ARCR5'	1.452	0.157	1.000	'ARCR5'	1.384	0.176	1.000	0.475	0.724
'ARCR6'	0.779	0.442	1.000	'ARCR6'	0.210	0.835	1.000	0.816	0.235
'ARCR7'	-0.746	0.461	1.000	'ARCR7'	0.256	0.799	1.000	0.427	0.805
'ARCR8'	0.225	0.823	1.000	'ARCR8'	0.437	0.665	1.000	0.013	-2.646
'ARCR9'	1.448	0.158	1.000	'ARCR9'	1.088	0.285	1.000	0.360	-0.930
'CCbody1'	-0.006	0.996	1.000	'CCbody1'	0.875	0.389	1.000	0.260	1.149
'CCbody2'	2.358	0.025	0.888	'CCbody2'	2.788	0.009	0.568	0.211	1.278
'CCbody3'	2.340	0.026	0.896	'CCbody3'	3.245	0.003	0.245	0.976	-0.030
'CCbody4'	-1.749	0.090	0.999	'CCbody4'	-2.231	0.033	0.956	0.029	-2.291
'CCbody5'	-0.363	0.719	1.000	'CCbody5'	-0.030	0.976	1.000	0.159	-1.443
'CCbody6'	-0.572	0.571	1.000	'CCbody6'	-0.122	0.904	1.000	0.785	0.276
'CCbody7'	-0.718	0.478	1.000	'CCbody7'	-0.330	0.744	1.000	0.764	-0.303
'CCbody8'	1.140	0.263	1.000	'CCbody8'	-0.110	0.913	1.000	0.807	-0.246
'CCbody9'	1.983	0.056	0.990	'CCbody9'	0.970	0.340	1.000	0.661	0.443
'CCgenu1'	0.757	0.454	1.000	'CCgenu1'	1.240	0.224	1.000	0.545	-0.613
'CCgenu2'	-0.501	0.620	1.000	'CCgenu2'	-0.081	0.936	1.000	0.178	-1.378
'CCgenu3'	0.281	0.780	1.000	'CCgenu3'	0.175	0.862	1.000	0.971	0.037
'CCgenu4'	-1.009	0.321	1.000	'CCgenu4'	-0.978	0.336	1.000	0.198	-1.317
'CCgenu5'	1.360	0.184	1.000	'CCgenu5'	1.829	0.077	0.998	0.134	1.538
'CCgenu6'	-1.590	0.122	1.000	'CCgenu6'	-1.781	0.085	0.999	0.803	0.252
'CCgenu7'	0.772	0.446	1.000	'CCgenu7'	0.811	0.423	1.000	0.031	2.270
'CCgenu8'	0.055	0.956	1.000	'CCgenu8'	0.984	0.333	1.000	0.659	0.445
'CCgenu9'	-0.643	0.525	1.000	'CCgenu9'	-0.833	0.411	1.000	0.514	0.661
'CCgenu10'	-0.694	0.493	1.000	'CCgenu10'	-0.452	0.654	1.000	0.433	-0.795
'CCgenu11'	2.060	0.048	0.982	'CCgenu11'	0.640	0.527	1.000	0.128	1.565
'CCgenu12'	0.024	0.981	1.000	'CCgenu12'	-0.038	0.970	1.000	0.786	0.274
'CCsplen1'	-0.295	0.770	1.000	'CCsplen1'	-0.583	0.564	1.000	0.752	-0.319
'CCsplen2'	-0.370	0.714	1.000	'CCsplen2'	-0.256	0.799	1.000	0.497	-0.687
'CCsplen3'	1.599	0.120	1.000	'CCsplen3'	0.705	0.486	1.000	0.794	0.263
'CCsplen4'	-0.637	0.529	1.000	'CCsplen4'	-1.427	0.164	1.000	0.701	0.388
'CCsplen5'	0.617	0.542	1.000	'CCsplen5'	0.850	0.402	1.000	0.076	1.835
'CCsplen6'	0.483	0.632	1.000	'CCsplen6'	0.431	0.669	1.000	0.209	1.284
'CCsplen7'	1.344	0.189	1.000	'CCsplen7'	0.945	0.352	1.000	0.715	-0.369
'CCsplen8'	-1.281	0.210	1.000	'CCsplen8'	-1.589	0.122	1.000	0.280	-1.099
'CCsplen9'	1.481	0.149	1.000	'CCsplen9'	2.416	0.022	0.852	0.135	1.538
'CCsplen10'	-0.121	0.904	1.000	'CCsplen10'	-0.481	0.634	1.000	0.946	0.069
'Cing_L1'	-0.631	0.533	1.000	'Cing_L1'	-0.822	0.417	1.000	0.845	0.197
'Cing_L2'	0.250	0.804	1.000	'Cing_L2'	0.537	0.595	1.000	0.328	-0.995
'Cing_L3'	4.195	0.000	0.026	'Cing_L3'	4.060	0.000	0.035	0.169	1.408
'Cing_L4'	-0.848	0.403	1.000	'Cing_L4'	-1.064	0.296	1.000	0.501	0.680
'Cing_L5'	-0.586	0.562	1.000	'Cing_L5'	-0.776	0.444	1.000	0.084	-1.786
'Cing_L6'	1.890	0.068	0.997	'Cing_L6'	1.022	0.315	1.000	0.686	-0.409
'Cing_L7'	1.542	0.133	1.000	'Cing_L7'	-0.134	0.894	1.000	0.944	-0.071
'Cing_L8'	-0.563	0.577	1.000	'Cing_L8'	-1.182	0.246	1.000	0.302	-1.050
'Cing_L9'	0.009	0.993	1.000	'Cing_L9'	-0.126	0.900	1.000	0.713	-0.371
'Cing_R1'	0.043	0.966	1.000	'Cing_R1'	0.556	0.583	1.000	0.879	-0.154
'Cing_R2'	1.298	0.204	1.000	'Cing_R2'	1.207	0.236	1.000	0.429	0.801
'Cing_R3'	-3.601	0.001	0.111	'Cing_R3'	-5.604	0.000	0.001	0.062	-1.940
'Cing_R4'	-1.082	0.288	1.000	'Cing_R4'	-1.318	0.197	1.000	0.211	-1.277
'Cing_R5'	-0.201	0.842	1.000	'Cing_R5'	-0.742	0.463	1.000	0.333	0.985
'Cing_R6'	-2.137	0.041	0.977	'Cing_R6'	-1.855	0.073	0.998	0.737	-0.339
'Cing_R7'	0.799	0.430	1.000	'Cing_R7'	1.825	0.078	0.998	0.067	1.899
'Cing_R8'	0.099	0.922	1.000	'Cing_R8'	0.324	0.748	1.000	0.477	0.720
'Cing_R9'	-1.406	0.170	1.000	'Cing_R9'	-1.859	0.073	0.998	0.237	-1.206
'Cing_R10'	-1.100	0.280	1.000	'Cing_R10'	-0.672	0.506	1.000	0.728	0.351
'FORNIX_left1'	0.468	0.643	1.000	'FORNIX_left1'	0.099	0.922	1.000	0.228	1.231
'FORNIX_left2'	-1.098	0.281	1.000	'FORNIX_left2'	-0.396	0.695	1.000	0.639	-0.474
'FORNIX_left3'	-0.341	0.736	1.000	'FORNIX_left3'	-0.975	0.337	1.000	0.812	-0.240
'FORNIX_left4'	-0.025	0.980	1.000	'FORNIX_left4'	0.566	0.575	1.000	0.204	1.299
'FORNIX_left5'	-1.494	0.146	1.000	'FORNIX_left5'	-1.040	0.307	1.000	0.276	-1.109
'FORNIX_left6'	-2.428	0.021	0.871	'FORNIX_left6'	-0.372	0.713	1.000	0.751	-0.320
'FORNIX_left7'	-2.064	0.048	0.987	'FORNIX_left7'	-0.407	0.687	1.000	0.577	-0.563
'FORNIX_right1'	0.044	0.965	1.000	'FORNIX_right1'	-0.782	0.441	1.000	0.256	-1.161
'FORNIX_right2'	-0.448	0.657	1.000	'FORNIX_right2'	-0.767	0.449	1.000	0.089	-1.760
'FORNIX_right3'	-2.474	0.019	0.840	'FORNIX_right3'	-1.094	0.283	1.000	0.479	0.717
'FORNIX_right4'	1.373	0.180	1.000	'FORNIX_right4'	0.881	0.386	1.000	0.870	0.165
'FORNIX_right5'	-1.915	0.065	0.997	'FORNIX_right5'	-1.154	0.258	1.000	0.730	-0.349
'FORNIX_right6'	-0.165	0.870	1.000	'FORNIX_right6'	-0.251	0.804	1.000	0.583	-0.556
'FORNIX_right7'	-1.713	0.097	1.000	'FORNIX_right7'	-0.743	0.463	1.000	0.347	0.957
'IFOL1'	0.379	0.708	1.000	'IFOL1'	0.307	0.761	1.000	0.283	1.093
'IFOL2'	-0.067	0.947	1.000	'IFOL2'	0.251	0.803	1.000	0.346	-0.958
'IFOL3'	2.027	0.051	0.986	'IFOL3'	1.601	0.119	1.000	0.609	0.517
'IFOL4'	-0.025	0.980	1.000	'IFOL4'	-0.577	0.568	1.000	0.979	-0.027
'IFOL5'	0.734	0.468	1.000	'IFOL5'	0.628	0.535	1.000	0.233	1.218
'IFOL6'	-1.177	0.248	1.000	'IFOL6'	-0.784	0.439	1.000	0.190	-1.340
'IFOL7'	-0.187	0.853	1.000	'IFOL7'	0.646	0.523	1.000	0.564	-0.583
'IFOL8'	1.016	0.317	1.000	'IFOL8'	1.102	0.279	1.000	0.067	1.904
'IFOL9'	-0.086	0.932	1.000	'IFOL9'	0.078	0.938	1.000	0.251	1.172
'IFOL10'	-0.018	0.986	1.000	'IFOL10'	-0.047	0.963	1.000	0.178	-1.379
'IFOL11'	-0.589	0.560	1.000	'IFOL11'	-0.633	0.531	1.000	0.693	-0.398
'IFOL12'	0.429	0.671	1.000	'IFOL12'	1.075	0.291	1.000	0.534	-0.629
'IFOL13'	0.219	0.828	1.000	'IFOL13'	-1.394	0.173	1.000	0.463	-0.743
'IFOL14'	1.056	0.299	1.000	'IFOL14'	1.123	0.270	1.000	0.046	2.081
'IFOR1'	0.641	0.526	1.000	'IFOR1'	0.411	0.684	1.000	0.944	0.071
'IFOR2'	-0.114	0.910	1.000	'IFOR2'	0.109	0.914	1.000	0.324	1.003
'IFOR3'	1.460	0.154	1.000	'IFOR3'	1.170	0.251	1.000	0.928	0.091
'IFOR4'	-1.095	0.282	1.000	'IFOR4'	-0.505	0.617	1.000	0.130	-1.558
'IFOR5'	0.663	0.512	1.000	'IFOR5'	-0.203	0.841	1.000	0.484	-0.708
'IFOR6'	0.641	0.527	1.000	'IFOR6'	0.174	0.863	1.000	0.175	-1.388
'IFOR7'	0.042	0.967	1.000	'IFOR7'	0.591	0.559	1.000	0.680	0.416
'IFOR8'	1.026	0.313	1.000	'IFOR8'	1.358	0.184	1.000	0.471	0.730
'IFOR9'	-0.198	0.844	1.000	'IFOR9'	-1.123	0.270	1.000	0.131	-1.553
'IFOR10'	0.616	0.543	1.000	'IFOR10'	0.141	0.889	1.000	0.616	0.507
'IFOR11'	-1.485	0.148	1.000	'IFOR11'	-0.822	0.418	1.000	0.509	0.668
'IFOR12'	1.564	0.128	1.000	'IFOR12'	1.209	0.236	1.000	0.525	0.643
'IFOR13'	1.785	0.084	1.000	'IFOR13'	1.445	0.158	1.000	0.845	0.197

'IFOR14'	-1.483	0.148	1.000	'IFOR14'	-0.836	0.409	1.000	0.391	-0.870
'IFOR15'	0.654	0.518	1.000	'IFOR15'	-0.096	0.924	1.000	0.625	0.494
'PHCL1'	-0.537	0.595	1.000	'PHCL1'	-0.756	0.455	1.000	0.476	-0.721
'PHCL2'	1.058	0.298	1.000	'PHCL2'	1.102	0.279	1.000	0.890	0.139
'PHCL3'	1.044	0.305	1.000	'PHCL3'	0.589	0.560	1.000	0.967	0.042
'PHCL4'	-1.179	0.247	1.000	'PHCL4'	-1.431	0.162	1.000	0.128	-1.567
'PHCL5'	-0.085	0.933	1.000	'PHCL5'	1.167	0.252	1.000	0.584	-0.554
'PHCL6'	1.444	0.159	1.000	'PHCL6'	1.580	0.124	1.000	0.701	0.388
'PHCL7'	0.358	0.723	1.000	'PHCL7'	1.168	0.252	1.000	0.391	0.870
'PHCL8'	-1.244	0.223	1.000	'PHCL8'	-2.223	0.034	0.957	0.243	-1.192
'PHCL9'	-0.039	0.969	1.000	'PHCL9'	-0.431	0.669	1.000	0.898	0.129
'PHCL10'	0.434	0.667	1.000	'PHCL10'	0.786	0.438	1.000	0.221	1.249
'PHCR1'	0.893	0.379	1.000	'PHCR1'	1.243	0.223	1.000	0.907	0.118
'PHCR2'	-0.431	0.670	1.000	'PHCR2'	0.172	0.865	1.000	0.481	-0.713
'PHCR3'	-0.438	0.665	1.000	'PHCR3'	-0.452	0.654	1.000	0.806	0.247
'PHCR4'	0.158	0.876	1.000	'PHCR4'	-0.544	0.590	1.000	0.075	-1.843
'PHCR5'	-1.312	0.199	1.000	'PHCR5'	-2.160	0.039	0.975	0.804	-0.250
'PHCR6'	-0.507	0.616	1.000	'PHCR6'	-0.604	0.550	1.000	0.723	0.358
'PHCR7'	1.039	0.307	1.000	'PHCR7'	2.163	0.038	0.962	0.163	1.432
'PHCR8'	-0.465	0.645	1.000	'PHCR8'	0.306	0.762	1.000	0.531	0.634
'PHCR9'	-0.239	0.813	1.000	'PHCR9'	-0.006	0.995	1.000	0.481	0.713
'PHCR10'	-3.084	0.004	0.343	'PHCR10'	-3.696	0.001	0.091	0.330	-0.989
'UNCL1'	0.621	0.539	1.000	'UNCL1'	0.405	0.688	1.000	0.472	0.728
'UNCL2'	0.255	0.800	1.000	'UNCL2'	0.327	0.746	1.000	0.621	0.499
'UNCL3'	0.433	0.668	1.000	'UNCL3'	0.198	0.845	1.000	0.546	-0.611
'UNCL4'	0.143	0.888	1.000	'UNCL4'	0.044	0.966	1.000	0.981	-0.024
'UNCL5'	0.687	0.497	1.000	'UNCL5'	-0.040	0.969	1.000	0.853	0.187
'UNCL6'	0.542	0.592	1.000	'UNCL6'	0.854	0.400	1.000	0.438	-0.787
'UNCL7'	-0.172	0.865	1.000	'UNCL7'	-0.099	0.922	1.000	0.219	-1.255
'UNCL8'	-0.994	0.328	1.000	'UNCL8'	-0.504	0.618	1.000	0.127	-1.570
'UNCL9'	-1.344	0.189	1.000	'UNCL9'	-1.703	0.099	1.000	0.967	0.042
'UNCR1'	0.053	0.958	1.000	'UNCR1'	0.632	0.532	1.000	0.735	-0.341
'UNCR2'	0.688	0.497	1.000	'UNCR2'	1.135	0.265	1.000	0.006	2.937
'UNCR3'	-0.551	0.585	1.000	'UNCR3'	0.073	0.942	1.000	0.681	0.415
'UNCR4'	1.182	0.246	1.000	'UNCR4'	1.631	0.113	1.000	0.056	1.988
'UNCR5'	-0.692	0.494	1.000	'UNCR5'	-0.794	0.433	1.000	0.666	0.436
'UNCR6'	2.025	0.052	0.986	'UNCR6'	2.250	0.032	0.937	0.469	0.734
'UNCR7'	0.131	0.896	1.000	'UNCR7'	0.530	0.600	1.000	0.509	0.669
'UNCR8'	1.273	0.212	1.000	'UNCR8'	0.543	0.591	1.000	0.442	0.779
'UNCR9'	-0.980	0.335	1.000	'UNCR9'	-0.193	0.848	1.000	0.534	-0.629
'corticoSpinalL1'	-1.260	0.217	1.000	'corticoSpinalL1'	-0.513	0.612	1.000	0.590	-0.544
'corticoSpinalL2'	-0.266	0.792	1.000	'corticoSpinalL2'	0.836	0.409	1.000	0.302	1.050
'corticoSpinalL3'	2.188	0.036	0.955	'corticoSpinalL3'	1.891	0.068	0.996	0.941	-0.075
'corticoSpinalL4'	2.606	0.014	0.720	'corticoSpinalL4'	3.348	0.002	0.195	0.162	1.435
'corticoSpinalL5'	-0.880	0.386	1.000	'corticoSpinalL5'	-1.670	0.105	1.000	0.647	-0.463
'corticoSpinalL6'	2.064	0.047	0.981	'corticoSpinalL6'	2.193	0.036	0.955	0.761	-0.307
'corticoSpinalL7'	0.626	0.536	1.000	'corticoSpinalL7'	0.190	0.850	1.000	0.608	0.518
'corticoSpinalL8'	1.266	0.215	1.000	'corticoSpinalL8'	1.908	0.066	0.995	0.080	1.810
'corticoSpinalL9'	1.064	0.295	1.000	'corticoSpinalL9'	1.001	0.325	1.000	0.239	1.201
'corticoSpinalR1'	-1.740	0.092	0.999	'corticoSpinalR1'	-0.943	0.353	1.000	0.798	0.259
'corticoSpinalR2'	-0.793	0.434	1.000	'corticoSpinalR2'	-0.455	0.652	1.000	0.274	-1.113
'corticoSpinalR3'	3.404	0.002	0.171	'corticoSpinalR3'	2.881	0.007	0.490	0.906	0.119
'corticoSpinalR4'	2.312	0.028	0.911	'corticoSpinalR4'	2.733	0.010	0.613	0.986	0.017
'corticoSpinalR5'	1.905	0.066	0.997	'corticoSpinalR5'	1.513	0.141	1.000	0.584	0.553
'corticoSpinalR6'	0.255	0.801	1.000	'corticoSpinalR6'	0.368	0.715	1.000	0.619	-0.503
'corticoSpinalR7'	1.461	0.154	1.000	'corticoSpinalR7'	0.651	0.520	1.000	0.645	-0.465
'corticoSpinalR8'	1.592	0.121	1.000	'corticoSpinalR8'	2.097	0.044	0.976	0.193	1.332
'corticoSpinalR9'	1.905	0.066	0.997	'corticoSpinalR9'	2.335	0.026	0.894	0.863	0.174
'corticoSpinalR10'	-1.525	0.137	1.000	'corticoSpinalR10'	-2.147	0.040	0.978	0.275	-1.113
'ilfLeft1'	-0.212	0.834	1.000	'ilfLeft1'	0.535	0.596	1.000	0.612	-0.512
'ilfLeft2'	0.744	0.463	1.000	'ilfLeft2'	1.930	0.063	0.993	0.141	1.512
'ilfLeft3'	-0.772	0.446	1.000	'ilfLeft3'	-0.819	0.419	1.000	0.970	0.038
'ilfLeft4'	-1.188	0.244	1.000	'ilfLeft4'	-0.728	0.472	1.000	0.427	-0.805
'ilfLeft5'	-0.312	0.757	1.000	'ilfLeft5'	-0.435	0.666	1.000	0.086	-1.777
'ilfLeft6'	0.830	0.413	1.000	'ilfLeft6'	1.138	0.264	1.000	0.133	1.544
'ilfLeft7'	1.207	0.237	1.000	'ilfLeft7'	1.037	0.308	1.000	0.242	1.193
'ilfLeft8'	-0.257	0.799	1.000	'ilfLeft8'	0.227	0.822	1.000	0.545	0.612
'ilfLeft9'	0.778	0.442	1.000	'ilfLeft9'	1.677	0.104	1.000	0.344	-0.962
'ilfLeft10'	-0.048	0.962	1.000	'ilfLeft10'	-0.711	0.483	1.000	0.942	-0.074
'ilfLeft11'	-0.811	0.423	1.000	'ilfLeft11'	-0.354	0.726	1.000	0.219	-1.257
'ilfLeft12'	-0.242	0.811	1.000	'ilfLeft12'	0.223	0.825	1.000	0.395	0.863
'ilfRight1'	-0.031	0.975	1.000	'ilfRight1'	0.040	0.968	1.000	0.845	-0.197
'ilfRight2'	0.686	0.498	1.000	'ilfRight2'	0.553	0.584	1.000	0.085	-1.783
'ilfRight3'	1.946	0.061	0.994	'ilfRight3'	1.579	0.125	1.000	0.010	2.751
'ilfRight4'	1.838	0.076	0.999	'ilfRight4'	1.620	0.115	1.000	0.049	2.055
'ilfRight5'	0.632	0.532	1.000	'ilfRight5'	0.399	0.693	1.000	0.396	-0.862
'ilfRight6'	2.423	0.021	0.848	'ilfRight6'	1.882	0.069	0.997	0.805	0.249
'ilfRight7'	0.362	0.720	1.000	'ilfRight7'	0.042	0.966	1.000	0.356	0.937
'ilfRight8'	0.125	0.901	1.000	'ilfRight8'	0.180	0.859	1.000	0.338	-0.975
'ilfRight9'	-1.314	0.198	1.000	'ilfRight9'	-1.041	0.306	1.000	0.816	0.235
'ilfRight10'	-0.255	0.800	1.000	'ilfRight10'	-0.431	0.669	1.000	0.726	-0.354
'ilfRight11'	-0.828	0.414	1.000	'ilfRight11'	-0.392	0.698	1.000	0.432	-0.796
'ilfRight12'	0.053	0.958	1.000	'ilfRight12'	-0.560	0.580	1.000	0.424	-0.811

GREY MATTER MORPHOLOGY FINDINGS FOR PEN_SZ

T

	'surf_area'	'gm_vol'	'thick_avg'	'curv_mean'	'curv_gauss'	'fold_idx'	'curv_idx'
'lh.aparc_bankssts '	0.730671031	0.837	0.590	0.538	0.509	0.065	0.565
'lh.aparc_caudalanteriorcingulate '	0.205	0.316	0.745	0.775	0.828	0.412	0.830
'lh.aparc_caudalmiddlefrontal '	0.528	0.520	0.483	0.702	-0.295	-0.451	0.382
'lh.aparc_cuneus '	0.002	0.297	0.942	0.774	0.877	0.195	0.627
'lh.aparc_entorhinal '	1.167	1.011	0.642	0.704	1.390	-0.432	1.877
'lh.aparc_fusiform '	-0.086	0.111	0.711	0.706	1.820	0.357	1.431
'lh.aparc_inferiorparietal '	0.392	0.531	0.715	0.659	-0.324	-0.435	1.556
'lh.aparc_inferiortemporal '	0.320	0.444	0.678	0.714	-0.587	-1.280	1.296
'lh.aparc_isthmuscingulate '	0.352	0.337	0.573	0.550	-0.339	-0.134	-0.667
'lh.aparc_lateraloccipital '	0.397	0.849	0.995	0.690	0.708	0.170	1.389
'lh.aparc_lateralorbitofrontal '	-0.189	-0.099	0.494	0.586	0.454	0.933	1.401
'lh.aparc_lingual '	0.174	0.618	0.847	0.789	1.034	1.705	1.962
'lh.aparc_medialorbitofrontal '	0.345	0.786	0.707	0.688	1.976	-0.501	3.671
'lh.aparc_middletemporal '	0.594	0.620	0.618	0.802	1.646	0.513	0.946
'lh.aparc_parahippocampal '	0.241	0.048	0.468	0.512	-0.748	-0.913	-0.770
'lh.aparc_paracentral '	0.325	0.436	0.617	0.580	-1.269	-0.826	-1.103
'lh.aparc_parsopercularis '	-0.131	-0.012	0.610	0.718	0.723	0.246	0.278
'lh.aparc_parsorbitalis '	-0.059	0.139	0.495	0.672	0.797	0.795	0.222
'lh.aparc_parstriangularis '	0.249	0.461	0.639	0.843	0.387	0.624	0.863
'lh.aparc_pericalcarine '	-0.218	-0.359	0.661	0.511	1.166	1.091	0.309
'lh.aparc_postcentral '	0.243	0.326	0.668	0.791	1.709	1.863	0.052
'lh.aparc_posteriorcingulate '	0.462	0.553	0.670	1.069	0.907	1.443	0.936
'lh.aparc_precentral '	0.551	0.430	0.465	0.732	-0.856	-0.945	-0.283
'lh.aparc_precuneus '	-0.092	0.077	0.793	0.619	0.186	-0.126	-0.025
'lh.aparc_rostralanteriorcingulate'	0.159	0.470	0.821	1.127	0.917	0.748	1.009
'lh.aparc_rostralmiddlefrontal '	0.488	0.551	0.482	0.595	2.070	0.739	1.370
'lh.aparc_superiorfrontal '	-0.009	0.221	0.658	0.795	0.380	-0.765	0.436
'lh.aparc_superiorparietal '	0.372	0.345	0.656	0.806	0.524	0.642	0.082
'lh.aparc_superiortemporal '	0.459	0.293	0.348	0.800	-0.291	0.008	0.936
'lh.aparc_supramarginal '	-0.205	-0.170	0.599	0.803	0.668	0.100	-0.442
'lh.aparc_frontalpole '	-0.010	0.532	0.758	1.191	1.183	1.086	1.876
'lh.aparc_temporalpole '	0.218	-0.083	0.378	0.597	0.766	0.934	0.701
'lh.aparc_transversetemporal '	0.628	0.640	0.504	0.309	-0.935	-0.314	-0.796
'lh.aparc_insula '	0.182	0.396	0.720	0.512	-0.475	-0.507	-0.357

	'surf_area'	'gm_vol'	'thick_avg'	'curv_mean'	'curv_gauss'	'fold_idx'	'curv_idx'
'rh.aparc_bankssts '	0.299	0.387	0.681	0.667	0.743	0.107	0.056
'rh.aparc_caudalanteriorcingulate '	0.112	0.088	0.574	0.793	1.362	1.217	1.254
'rh.aparc_caudalmiddlefrontal '	0.419	0.411	0.659	0.938	0.094	-0.281	-0.067
'rh.aparc_cuneus '	0.475	0.691	0.803	0.631	0.449	0.531	0.535
'rh.aparc_entorhinal '	0.497	0.227	0.398	0.699	1.277	0.179	0.860
'rh.aparc_fusiform '	0.111	0.467	0.838	0.736	0.449	-0.254	0.611
'rh.aparc_inferiorparietal '	0.704	0.858	0.705	0.801	0.694	0.252	1.253
'rh.aparc_inferiortemporal '	0.040	0.086	0.686	0.727	0.163	0.566	0.545
'rh.aparc_isthmuscingulate '	0.042	0.241	0.825	0.502	-0.798	-0.419	0.381
'rh.aparc_lateraloccipital '	0.385	0.740	0.916	0.697	1.128	0.637	0.405
'rh.aparc_lateralorbitofrontal '	0.073	0.359	0.745	0.670	-0.007	0.237	0.567
'rh.aparc_lingual '	0.054	0.733	1.256	0.798	-0.125	-0.975	0.215
'rh.aparc_medialorbitofrontal '	0.082	0.548	1.029	0.630	0.554	-0.815	0.385
'rh.aparc_middletemporal '	0.172	0.442	0.670	0.667	0.698	-0.439	0.201
'rh.aparc_parahippocampal '	0.105	-0.154	0.499	0.931	0.518	1.977	-0.286
'rh.aparc_paracentral '	-0.046	0.023	0.706	0.440	-0.974	-0.880	-1.015
'rh.aparc_parsopercularis '	0.574	0.783	0.652	0.774	0.090	0.147	0.840
'rh.aparc_parsorbitalis '	0.232	0.729	0.871	0.792	1.166	0.844	1.516
'rh.aparc_parstriangularis '	0.534	0.772	0.672	0.772	0.822	0.568	1.308
'rh.aparc_pericalcarine '	0.161	0.513	0.884	1.719	0.653	0.496	2.097
'rh.aparc_postcentral '	0.515	0.467	0.474	0.792	1.545	1.096	1.040
'rh.aparc_posteriorcingulate '	0.306	0.247	0.672	0.413	-1.136	-1.516	-0.843
'rh.aparc_precentral '	0.357	0.261	0.531	0.802	1.645	1.716	0.177
'rh.aparc_precuneus '	0.017	0.217	0.890	0.785	-0.417	0.082	0.272
'rh.aparc_rostralanteriorcingulate'	-0.209	-0.312	0.821	0.747	0.194	-0.488	-0.344
'rh.aparc_rostralmiddlefrontal '	0.063	0.234	0.703	0.764	0.917	0.083	0.632
'rh.aparc_superiorfrontal '	-0.053	0.068	0.600	0.738	0.565	-0.490	0.001
'rh.aparc_superiorparietal '	0.014	0.114	0.702	0.811	0.841	0.638	0.352
'rh.aparc_superiortemporal '	0.310	0.450	0.652	0.896	1.280	1.517	0.580
'rh.aparc_supramarginal '	-0.031	0.055	0.655	0.661	-0.110	-0.408	0.156
'rh.aparc_frontalpole '	0.661	1.019	0.721	0.592	0.540	0.359	0.477
'rh.aparc_temporalpole '	0.046	0.130	0.552	1.167	0.892	0.676	0.920
'rh.aparc_transversetemporal '	0.565	0.415	0.420	0.585	-0.140	-0.146	-0.263
'rh.aparc_insula '	0.299	0.431	0.754	0.555	-0.200	0.146	-0.090

P

'lh.aparc_bankssts '	0.470	0.409	0.559	0.594	0.614	0.948	0.576
'lh.aparc_caudalanteriorcingulate '	0.839	0.754	0.461	0.444	0.414	0.683	0.412
'lh.aparc_caudalmiddlefrontal '	0.601	0.607	0.633	0.488	0.770	0.655	0.705
'lh.aparc_cuneus '	0.999	0.769	0.353	0.445	0.387	0.847	0.535
'lh.aparc_entorhinal '	0.252	0.319	0.525	0.486	0.174	0.668	0.069
'lh.aparc_fusiform '	0.932	0.913	0.482	0.485	0.078	0.723	0.162
'lh.aparc_inferiorparietal '	0.698	0.599	0.480	0.514	0.748	0.667	0.129
'lh.aparc_inferiortemporal '	0.751	0.660	0.503	0.480	0.561	0.209	0.204
'lh.aparc_isthmuscingulate '	0.727	0.738	0.570	0.586	0.737	0.895	0.510
'lh.aparc_lateraloccipital '	0.694	0.402	0.327	0.495	0.484	0.866	0.174
'lh.aparc_lateralorbitofrontal '	0.851	0.922	0.624	0.562	0.653	0.358	0.171
'lh.aparc_lingual '	0.863	0.541	0.403	0.436	0.309	0.098	0.058
'lh.aparc_medialorbitofrontal '	0.733	0.438	0.484	0.496	0.057	0.620	0.001
'lh.aparc_middletemporal '	0.557	0.540	0.541	0.428	0.109	0.611	0.351
'lh.aparc_parahippocampal '	0.811	0.962	0.643	0.612	0.460	0.368	0.447
'lh.aparc_paracentral '	0.747	0.666	0.541	0.566	0.213	0.415	0.278
'lh.aparc_parsopercularis '	0.896	0.990	0.546	0.478	0.475	0.807	0.783
'lh.aparc_parsorbitalis '	0.953	0.891	0.624	0.506	0.431	0.432	0.825
'lh.aparc_parstriangularis '	0.805	0.648	0.527	0.405	0.701	0.537	0.394
'lh.aparc_pericalcarine '	0.829	0.722	0.513	0.613	0.252	0.283	0.759
'lh.aparc_postcentral '	0.810	0.746	0.509	0.435	0.097	0.071	0.959
'lh.aparc_posteriorcingulate '	0.647	0.584	0.508	0.293	0.371	0.159	0.356
'lh.aparc_precentral '	0.586	0.670	0.645	0.469	0.398	0.352	0.779
'lh.aparc_precuneus '	0.927	0.939	0.434	0.540	0.854	0.900	0.981
'lh.aparc_rostralanteriorcingulate'	0.874	0.641	0.418	0.268	0.366	0.460	0.320
'lh.aparc_rostralmiddlefrontal '	0.629	0.586	0.633	0.556	0.046	0.465	0.180
'lh.aparc_superiorfrontal '	0.993	0.826	0.515	0.432	0.706	0.450	0.666
'lh.aparc_superiorparietal '	0.712	0.732	0.517	0.426	0.604	0.525	0.935
'lh.aparc_superiortemporal '	0.649	0.771	0.730	0.429	0.773	0.993	0.356
'lh.aparc_supramarginal '	0.839	0.866	0.553	0.428	0.509	0.921	0.661
'lh.aparc_frontalpole '	0.992	0.598	0.454	0.242	0.245	0.285	0.069
'lh.aparc_temporalpole '	0.829	0.935	0.708	0.555	0.449	0.357	0.488
'lh.aparc_transversetemporal '	0.534	0.527	0.618	0.760	0.357	0.756	0.432
'lh.aparc_insula '	0.856	0.695	0.476	0.612	0.638	0.615	0.723

	'surf_area'	'gm_vol'	'thick_avg'	'curv_mean'	'curv_gauss'	'fold_idx'	'curv_idx'
'rh.aparc_bankssts '	0.767	0.701	0.501	0.509	0.463	0.915	0.956
'rh.aparc_caudalanteriorcingulate '	0.911	0.930	0.570	0.434	0.183	0.232	0.219
'rh.aparc_caudalmiddlefrontal '	0.678	0.683	0.514	0.355	0.926	0.780	0.947
'rh.aparc_cuneus '	0.638	0.494	0.428	0.532	0.656	0.599	0.596
'rh.aparc_entorhinal '	0.623	0.822	0.693	0.489	0.210	0.859	0.396
'rh.aparc_fusiform '	0.913	0.643	0.408	0.467	0.656	0.801	0.546
'rh.aparc_inferiorparietal '	0.486	0.397	0.486	0.429	0.493	0.803	0.219
'rh.aparc_inferiortemporal '	0.969	0.932	0.497	0.472	0.872	0.575	0.589
'rh.aparc_isthmuscingulate '	0.967	0.811	0.416	0.619	0.431	0.678	0.705
'rh.aparc_lateraloccipital '	0.702	0.464	0.366	0.491	0.268	0.528	0.688
'rh.aparc_lateralorbitofrontal '	0.942	0.722	0.462	0.508	0.995	0.814	0.575
'rh.aparc_lingual '	0.957	0.469	0.218	0.431	0.902	0.337	0.831
'rh.aparc_medialorbitofrontal '	0.935	0.587	0.311	0.533	0.583	0.421	0.703
'rh.aparc_middletemporal '	0.864	0.661	0.508	0.509	0.490	0.664	0.842
'rh.aparc_parahippocampal '	0.917	0.878	0.621	0.359	0.608	0.056	0.777
'rh.aparc_paracentral '	0.964	0.982	0.485	0.663	0.337	0.385	0.317
'rh.aparc_parsopercularis '	0.570	0.439	0.519	0.445	0.929	0.884	0.407
'rh.aparc_parsorbitalis '	0.818	0.471	0.390	0.434	0.252	0.405	0.139
'rh.aparc_parstriangularis '	0.597	0.446	0.506	0.446	0.417	0.574	0.200
'rh.aparc_pericalcarine '	0.873	0.611	0.383	0.095	0.518	0.623	0.044
'rh.aparc_postcentral '	0.610	0.643	0.638	0.434	0.132	0.281	0.306
'rh.aparc_posteriorcingulate '	0.762	0.807	0.506	0.682	0.264	0.139	0.405
'rh.aparc_precentral '	0.723	0.796	0.599	0.428	0.109	0.096	0.861
'rh.aparc_precuneus '	0.986	0.829	0.380	0.438	0.679	0.935	0.787
'rh.aparc_rostralanteriorcingulate'	0.836	0.757	0.417	0.460	0.848	0.629	0.733
'rh.aparc_rostralmiddlefrontal '	0.950	0.816	0.487	0.450	0.366	0.935	0.532
'rh.aparc_superiorfrontal '	0.958	0.946	0.553	0.465	0.576	0.628	0.999
'rh.aparc_superiorparietal '	0.989	0.910	0.487	0.423	0.407	0.528	0.727
'rh.aparc_superiortemporal '	0.758	0.655	0.519	0.377	0.210	0.139	0.566
'rh.aparc_supramarginal '	0.976	0.956	0.517	0.514	0.913	0.686	0.877
'rh.aparc_frontalpole '	0.513	0.316	0.476	0.558	0.593	0.722	0.636
'rh.aparc_temporalpole '	0.963	0.898	0.585	0.252	0.379	0.504	0.364
'rh.aparc_transversetemporal '	0.576	0.681	0.678	0.563	0.889	0.885	0.794
'rh.aparc_insula '	0.766	0.669	0.456	0.582	0.842	0.885	0.929

P_CORR

'lh.aparc_bankssts '	0.542	0.539	0.493	0.574	0.992	1.000	0.958
'lh.aparc_caudalanteriorcingulate '	0.723	0.720	0.438	0.482	0.978	0.996	0.930
'lh.aparc_caudalmiddlefrontal '	0.613	0.646	0.529	0.513	1.000	0.998	0.974
'lh.aparc_cuneus '	0.775	0.728	0.354	0.482	0.976	0.999	0.951
'lh.aparc_entorhinal '	0.374	0.472	0.476	0.513	0.918	0.999	0.658
'lh.aparc_fusiform '	0.744	0.780	0.450	0.513	0.818	0.996	0.801
'lh.aparc_inferiorparietal '	0.666	0.642	0.448	0.529	1.000	0.999	0.767
'lh.aparc_inferiortemporal '	0.689	0.673	0.465	0.509	0.992	0.767	0.837
'lh.aparc_isthmuscingulate '	0.678	0.713	0.498	0.569	1.000	1.000	0.922
'lh.aparc_lateraloccipital '	0.665	0.536	0.330	0.517	0.984	0.999	0.816
'lh.aparc_lateralorbitofrontal '	0.706	0.775	0.525	0.557	0.994	0.981	0.811
'lh.aparc_lingual '	0.730	0.613	0.397	0.476	0.966	0.880	0.626
'lh.aparc_medialorbitofrontal '	0.680	0.557	0.451	0.518	0.772	0.997	0.080
'lh.aparc_middletemporal '	0.593	0.612	0.483	0.472	0.861	0.996	0.910
'lh.aparc_parahippocampal '	0.712	0.796	0.534	0.583	0.963	0.947	0.887
'lh.aparc_paracentral '	0.687	0.678	0.483	0.558	0.713	0.968	0.719
'lh.aparc_parsopercularis '	0.726	0.806	0.486	0.507	0.983	0.998	0.982
'lh.aparc_parsorbitalis '	0.753	0.772	0.525	0.523	0.979	0.988	0.986
'lh.aparc_parstriangularis '	0.710	0.665	0.476	0.457	0.995	0.993	0.924
'lh.aparc_pericalcarine '	0.693	0.671	0.470	0.583	0.954	0.972	0.980
'lh.aparc_postcentral '	0.711	0.715	0.468	0.475	0.845	0.848	0.992
'lh.aparc_posteriorcingulate '	0.640	0.635	0.467	0.363	0.975	0.931	0.913
'lh.aparc_precentral '	0.608	0.680	0.535	0.501	0.930	0.940	0.991
'lh.aparc_precuneus '	0.742	0.789	0.417	0.544	0.998	1.000	0.993
'lh.aparc_rostralanteriorcingulate'	0.736	0.662	0.408	0.342	0.975	0.990	0.900
'lh.aparc_rostralmiddlefrontal '	0.627	0.636	0.529	0.553	0.743	0.990	0.821
'lh.aparc_superiorfrontal '	0.770	0.748	0.471	0.475	0.996	0.978	0.970
'lh.aparc_superiorparietal '	0.671	0.709	0.472	0.471	0.992	0.992	0.990
'lh.aparc_superiortemporal '	0.641	0.728	0.571	0.473	1.000	1.000	0.913
'lh.aparc_supramarginal '	0.700	0.747	0.490	0.471	0.986	1.000	0.977
'lh.aparc_frontalpole '	0.770	0.642	0.431	0.319	0.952	0.972	0.658
'lh.aparc_temporalpole '	0.719	0.782	0.562	0.552	0.981	0.981	0.943
'lh.aparc_transversetemporal '	0.582	0.606	0.521	0.649	0.897	1.000	0.878
'lh.aparc_insula '	0.729	0.691	0.447	0.583	0.998	0.997	0.987

	'surf_area'	'gm_vol'	'thick_avg'	'curv_mean'	'curv_gauss'	'fold_idx'	'curv_idx'
'rh.aparc_bankssts '	0.696	0.694	0.463	0.525	0.982	1.000	0.991
'rh.aparc_caudalanteriorcingulate '	0.753	0.785	0.497	0.475	0.925	0.962	0.848
'rh.aparc_caudalmiddlefrontal '	0.657	0.686	0.470	0.419	0.999	1.000	0.994
'rh.aparc_cuneus '	0.633	0.589	0.414	0.539	0.994	0.995	0.960
'rh.aparc_entorhinal '	0.625	0.746	0.556	0.514	0.938	0.999	0.924
'rh.aparc_fusiform '	0.754	0.663	0.400	0.500	0.994	1.000	0.953
'rh.aparc_inferiorparietal '	0.554	0.531	0.452	0.473	0.985	0.998	0.848
'rh.aparc_inferiortemporal '	0.773	0.786	0.460	0.504	0.999	0.995	0.959
'rh.aparc_isthmuscingulate '	0.773	0.741	0.407	0.586	0.950	0.999	0.974
'rh.aparc_lateraloccipital '	0.668	0.572	0.364	0.515	0.958	0.993	0.972
'rh.aparc_lateralorbitofrontal '	0.764	0.705	0.438	0.524	1.000	0.998	0.957
'rh.aparc_lingual '	0.770	0.573	0.231	0.473	1.000	0.927	0.986
'rh.aparc_medialorbitofrontal '	0.761	0.636	0.319	0.540	0.991	0.970	0.974
'rh.aparc_middletemporal '	0.730	0.674	0.467	0.525	0.985	0.999	0.987
'rh.aparc_parahippocampal '	0.756	0.755	0.523	0.423	0.992	0.820	0.991
'rh.aparc_paracentral '	0.757	0.802	0.452	0.609	0.879	0.956	0.770
'rh.aparc_parsopercularis '	0.601	0.558	0.473	0.482	0.999	0.999	0.928
'rh.aparc_parsorbitalis '	0.716	0.574	0.385	0.475	0.954	0.985	0.779
'rh.aparc_parstriangularis '	0.612	0.562	0.466	0.483	0.978	0.995	0.835
'rh.aparc_pericalcarine '	0.735	0.648	0.379	0.140	0.987	0.996	0.581
'rh.aparc_postcentral '	0.617	0.663	0.532	0.475	0.888	0.971	0.893
'rh.aparc_posteriorcingulate '	0.693	0.739	0.466	0.618	0.796	0.562	0.857
'rh.aparc_precentral '	0.675	0.735	0.514	0.472	0.861	0.876	0.988
'rh.aparc_precuneus '	0.777	0.749	0.375	0.478	0.999	1.000	0.982
'rh.aparc_rostralanteriorcingulate'	0.698	0.692	0.408	0.495	0.998	0.998	0.987
'rh.aparc_rostralmiddlefrontal '	0.767	0.744	0.453	0.487	0.975	1.000	0.951
'rh.aparc_superiorfrontal '	0.755	0.790	0.490	0.500	0.990	0.998	0.992
'rh.aparc_superiorparietal '	0.778	0.779	0.454	0.470	0.978	0.993	0.978
'rh.aparc_superiortemporal '	0.692	0.670	0.473	0.438	0.938	0.917	0.956
'rh.aparc_supramarginal '	0.765	0.793	0.472	0.529	1.000	0.999	0.989
'rh.aparc_frontalpole '	0.570	0.469	0.447	0.554	0.992	0.996	0.966
'rh.aparc_temporalpole '	0.773	0.774	0.506	0.328	0.975	0.992	0.916
'rh.aparc_transversetemporal '	0.602	0.685	0.550	0.557	1.000	1.000	0.992
'rh.aparc_insula '	0.695	0.680	0.433	0.568	1.000	0.999	0.994

GREY MATTER MORPHOLOGY FINDINGS FOR PEN_DD

T

	'surf_area'	'gm_vol'	'thick_avg'	'curv_mean'	'curv_gauss'	'fold_idx'	'curv_idx'
'lh.aparc_bankssts '	0.8780	1.1621	0.6961	0.6356	0.5094	-0.0838	0.6010
'lh.aparc_caudalanteriorcingulate '	0.6355	0.7024	0.7264	0.8368	1.1691	0.7934	1.4256
'lh.aparc_caudalmiddlefrontal '	0.9597	1.1041	0.5968	0.7541	-0.4977	-0.4093	0.7539
'lh.aparc_cuneus '	0.4279	0.7522	1.0459	0.7600	0.3625	-0.1132	0.5757
'lh.aparc_entorhinal '	1.4501	1.2246	0.5435	0.5657	0.5703	-0.1762	1.7765
'lh.aparc_fusiform '	0.2750	0.5345	0.7055	0.7193	2.2055	0.4899	2.0456
'lh.aparc_inferiorparietal '	0.4005	0.6603	0.8903	0.8003	-0.1052	-0.4889	1.9224
'lh.aparc_inferiortemporal '	0.7030	0.8893	0.6979	0.7641	-0.4495	-1.2235	1.6322
'lh.aparc_isthmuscingulate '	0.7716	0.7796	0.6878	0.4059	-0.1674	-0.2146	-0.6053
'lh.aparc_lateraloccipital '	0.7569	1.3634	1.1462	0.8564	0.9199	0.5614	1.8366
'lh.aparc_lateralorbitofrontal '	-0.0805	0.0814	0.5517	0.6760	0.1052	0.0964	0.9675
'lh.aparc_lingual '	0.4347	0.9936	0.9981	0.8017	0.9005	2.0155	2.2697
'lh.aparc_medialorbitofrontal '	0.6050	0.9951	0.6037	0.6352	0.8182	-0.6113	1.0495
'lh.aparc_middletemporal '	0.7203	0.8060	0.6585	0.7970	0.0853	-0.2136	1.2813
'lh.aparc_parahippocampal '	0.7252	0.4563	0.4520	0.6484	0.2358	0.1333	-0.0671
'lh.aparc_paracentral '	0.4162	0.4602	0.6511	0.6357	-1.0002	-0.8080	-0.9939
'lh.aparc_parsopercularis '	0.1142	0.3598	0.7466	0.7097	0.7050	0.0818	0.4322
'lh.aparc_parsorbitalis '	0.0916	0.4464	0.6460	0.8084	0.3084	0.3463	0.6465
'lh.aparc_parstriangularis '	0.2849	0.5563	0.7133	0.8705	0.2661	0.3289	0.7012
'lh.aparc_pericalcarine '	0.0007	-0.0796	0.8242	0.6648	1.0494	1.0321	0.1580
'lh.aparc_postcentral '	0.4314	0.5236	0.6547	0.8829	2.3174	2.0096	0.6414
'lh.aparc_posteriorcingulate '	0.7588	0.8693	0.7040	1.0431	0.6068	1.5725	0.7137
'lh.aparc_precentral '	0.6980	0.6371	0.5368	0.8799	-0.8725	-0.9116	0.2491
'lh.aparc_precuneus '	0.3143	0.5362	0.8141	0.6329	0.2016	-0.0414	0.3489
'lh.aparc_rostralanteriorcingulate'	0.4951	0.6906	0.7433	1.0192	0.6670	0.6902	1.3183
'lh.aparc_rostralmiddlefrontal '	0.8166	0.9909	0.5775	0.6761	2.3753	1.0223	1.9368
'lh.aparc_superiorfrontal '	0.2513	0.4838	0.6232	0.8700	0.5437	-0.7095	0.8649
'lh.aparc_superiorparietal '	0.6796	0.7601	0.7767	0.9134	0.2698	0.2994	0.7202
'lh.aparc_superiortemporal '	0.8604	0.8713	0.5248	0.9359	-0.4637	-0.2107	0.9720
'lh.aparc_supramarginal '	0.2280	0.3328	0.6932	0.8525	0.8374	0.6080	-0.0924
'lh.aparc_frontalpole '	0.1746	0.8202	0.8561	1.2122	1.4451	1.4722	2.5706
'lh.aparc_temporalpole '	0.2883	-0.0853	0.3277	0.8764	1.0380	1.0497	0.8178
'lh.aparc_transversetemporal '	1.1852	1.4115	0.6702	0.2909	-0.6322	0.0022	-0.4024
'lh.aparc_insula '	0.5074	0.7813	0.7998	0.7134	-0.2582	-0.2920	0.0711

	'surf_area'	'gm_vol'	'thick_avg'	'curv_mean'	'curv_gauss'	'fold_idx'	'curv_idx'
'rh.aparc_bankssts '	0.5847	0.7598	0.7407	0.8634	1.2060	0.5999	0.5703
'rh.aparc_caudalanteriorcingulate '	0.3278	0.3168	0.4957	0.9125	1.6142	1.3165	1.6114
'rh.aparc_caudalmiddlefrontal '	0.9660	1.0200	0.7110	1.0139	0.7463	0.7855	0.9413
'rh.aparc_cuneus '	0.7591	0.9199	0.8027	0.5689	0.1293	0.3384	0.5387
'rh.aparc_entorhinal '	1.2193	0.9383	0.5019	0.9108	0.9490	0.4320	1.4271
'rh.aparc_fusiform '	0.4302	0.7569	0.8077	0.6501	0.5735	-0.0868	0.8045
'rh.aparc_inferiorparietal '	0.9437	1.0629	0.7419	0.8271	0.5306	0.1638	0.9549
'rh.aparc_inferiortemporal '	0.4186	0.4978	0.6915	0.7784	0.3787	0.8067	1.0073
'rh.aparc_isthmuscingulate '	0.4484	0.6935	0.8976	0.4131	-0.8803	-0.4846	0.3578
'rh.aparc_lateraloccipital '	0.8212	1.2423	0.9455	0.7176	1.0543	1.1438	0.9797
'rh.aparc_lateralorbitofrontal '	0.2510	0.6476	0.7876	0.7705	0.0236	0.6717	1.1123
'rh.aparc_lingual '	0.4488	1.0991	1.1787	0.7416	-0.1924	-0.8861	0.7943
'rh.aparc_medialorbitofrontal '	0.3282	0.7430	0.9156	0.7237	0.4374	-0.8635	0.1977
'rh.aparc_middletemporal '	0.4837	0.8270	0.7292	0.7107	0.5472	-0.5154	0.2277
'rh.aparc_parahippocampal '	0.5607	0.3254	0.5412	0.8151	0.4961	2.5112	-0.3216
'rh.aparc_paracentral '	0.0065	0.0767	0.7058	0.5427	-0.6100	-0.6436	-0.6480
'rh.aparc_parsopercularis '	0.5974	0.8894	0.7639	0.7992	-0.2764	-0.1865	0.3290
'rh.aparc_parsorbitalis '	0.3655	1.0425	0.8695	0.9267	1.3063	1.0113	1.9583
'rh.aparc_parstriangularis '	0.7825	1.0722	0.7039	0.7620	0.4415	0.2527	1.3723
'rh.aparc_pericalcarine '	0.6521	1.0116	0.9437	2.0023	1.1564	0.9381	2.8324
'rh.aparc_postcentral '	0.6696	0.6406	0.5582	1.0088	1.9291	1.4152	1.5132
'rh.aparc_posteriorcingulate '	0.7175	0.7158	0.6670	0.5465	-0.5141	-1.0195	-0.0040
'rh.aparc_precentral '	0.5947	0.4884	0.5455	0.9607	2.1037	1.9758	0.4871
'rh.aparc_precuneus '	0.2536	0.5014	0.9261	0.7946	-0.5005	-0.2984	0.7071
'rh.aparc_rostralanteriorcingulate'	0.0524	0.0162	0.9046	0.8438	0.4714	-0.0029	0.1459
'rh.aparc_rostralmiddlefrontal '	0.5351	0.7535	0.6990	0.8211	1.1675	0.3560	1.3083
'rh.aparc_superiorfrontal '	0.0909	0.2633	0.6166	0.7741	0.7222	-0.1784	0.2872
'rh.aparc_superiorparietal '	0.2317	0.3717	0.7595	0.9253	0.4999	0.1946	0.6660
'rh.aparc_superiortemporal '	0.5938	0.7821	0.7417	0.9930	1.2767	1.8200	0.9045
'rh.aparc_supramarginal '	0.3457	0.5009	0.7339	0.7187	0.1492	-0.2722	0.3758
'rh.aparc_frontalpole '	0.9973	1.1118	0.6442	0.5900	0.5424	0.5089	0.6272
'rh.aparc_temporalpole '	0.3352	0.4677	0.5576	0.9938	0.6335	0.1300	0.7458
'rh.aparc_transversetemporal '	0.8449	0.8253	0.6037	0.6456	0.4160	0.2164	0.4362
'rh.aparc_insula '	0.6552	0.8799	0.8297	0.6912	-0.1477	0.3792	0.0895

P

'lh.aparc_bankssts '	0.386274505	0.253512448	0.491221185	0.529422751	0.613837391	0.933731746	0.551935277
'lh.aparc_caudalanteriorcingulate '	0.5295	0.4873	0.4727	0.4087	0.2507	0.4332	0.1634
'lh.aparc_caudalmiddlefrontal '	0.3442	0.2775	0.5547	0.4562	0.6220	0.6850	0.4562
'lh.aparc_cuneus '	0.6715	0.4573	0.3032	0.4526	0.7193	0.9105	0.5687
'lh.aparc_entorhinal '	0.1565	0.2294	0.5905	0.5754	0.5723	0.8612	0.0849
'lh.aparc_fusiform '	0.7850	0.5966	0.4854	0.4770	0.0345	0.6275	0.0488
'lh.aparc_inferiorparietal '	0.6913	0.5136	0.3798	0.4293	0.9169	0.6281	0.0632
'lh.aparc_inferiortemporal '	0.4870	0.3803	0.4901	0.4502	0.6560	0.2298	0.1122
'lh.aparc_isthmuscingulate '	0.4458	0.4412	0.4964	0.6875	0.8681	0.8314	0.5491
'lh.aparc_lateraloccipital '	0.4545	0.1820	0.2600	0.3979	0.3643	0.5783	0.0753
'lh.aparc_lateralorbitofrontal '	0.9363	0.9356	0.5849	0.5037	0.9168	0.9237	0.3403
'lh.aparc_lingual '	0.6666	0.3276	0.3255	0.4285	0.3744	0.0521	0.0299
'lh.aparc_medialorbitofrontal '	0.5493	0.3269	0.5502	0.5297	0.4191	0.5452	0.3016
'lh.aparc_middletemporal '	0.4764	0.4260	0.5148	0.4312	0.9326	0.8322	0.2090
'lh.aparc_parahippocampal '	0.4735	0.6512	0.6542	0.5212	0.8151	0.8948	0.9469
'lh.aparc_paracentral '	0.6800	0.6484	0.5195	0.5294	0.3245	0.4249	0.3275
'lh.aparc_parsopercularis '	0.9098	0.7213	0.4606	0.4829	0.4858	0.9353	0.6684
'lh.aparc_parsorbitalis '	0.9275	0.6582	0.5227	0.4247	0.7597	0.7313	0.5224
'lh.aparc_parstriangularis '	0.7775	0.5818	0.4807	0.3903	0.7918	0.7443	0.4881
'lh.aparc_pericalcarine '	0.9994	0.9370	0.4157	0.5108	0.3016	0.3095	0.8754
'lh.aparc_postcentral '	0.6690	0.6041	0.5172	0.3837	0.0268	0.0527	0.5257
'lh.aparc_posteriorcingulate '	0.4534	0.3910	0.4864	0.3045	0.5481	0.1254	0.4804
'lh.aparc_precentral '	0.4901	0.5285	0.5950	0.3853	0.3892	0.3686	0.8049
'lh.aparc_precuneus '	0.7553	0.5954	0.4214	0.5312	0.8414	0.9673	0.7294
'lh.aparc_rostralanteriorcingulate'	0.6238	0.4946	0.4625	0.3155	0.5094	0.4949	0.1965
'lh.aparc_rostralmiddlefrontal '	0.4200	0.3289	0.5675	0.5037	0.0235	0.3141	0.0614
'lh.aparc_superiorfrontal '	0.8032	0.6317	0.5374	0.3906	0.5903	0.4830	0.3934
'lh.aparc_superiorparietal '	0.5015	0.4526	0.4429	0.3677	0.7890	0.7665	0.4765
'lh.aparc_superiortemporal '	0.3958	0.3899	0.6032	0.3561	0.6459	0.8344	0.3381
'lh.aparc_supramarginal '	0.8210	0.7414	0.4931	0.4001	0.4084	0.5474	0.9269
'lh.aparc_frontalpole '	0.8624	0.4180	0.3981	0.2340	0.1579	0.1504	0.0149
'lh.aparc_temporalpole '	0.7750	0.9325	0.7452	0.3871	0.3068	0.3015	0.4193
'lh.aparc_transversetemporal '	0.2444	0.1674	0.5074	0.7730	0.5316	0.9982	0.6900
'lh.aparc_insula '	0.6152	0.4402	0.4295	0.4806	0.7978	0.7721	0.9437

'rh.aparc_bankssts '	0.5628	0.4527	0.4641	0.3941	0.2364	0.5527	0.5723
'rh.aparc_caudalanteriorcingulate '	0.7451	0.7534	0.6234	0.3681	0.1160	0.1971	0.1166
'rh.aparc_caudalmiddlefrontal '	0.3411	0.3151	0.4821	0.3180	0.4607	0.4378	0.3534
'rh.aparc_cuneus '	0.4532	0.3643	0.4279	0.5733	0.8979	0.7372	0.5937
'rh.aparc_entorhinal '	0.2314	0.3549	0.6191	0.3690	0.3495	0.6686	0.1630
'rh.aparc_fusiform '	0.6698	0.4545	0.4250	0.5201	0.5702	0.9313	0.4269
'rh.aparc_inferiorparietal '	0.3522	0.2956	0.4634	0.4141	0.5993	0.8709	0.3466
'rh.aparc_inferiortemporal '	0.6782	0.6219	0.4941	0.4419	0.7073	0.4256	0.3211
'rh.aparc_isthmuscingulate '	0.6568	0.4928	0.3759	0.6822	0.3850	0.6312	0.7228
'rh.aparc_lateraloccipital '	0.4174	0.2229	0.3513	0.4780	0.2994	0.2609	0.3343
'rh.aparc_lateralorbitofrontal '	0.8034	0.5217	0.4365	0.4465	0.9813	0.5064	0.2741
'rh.aparc_lingual '	0.6565	0.2797	0.2470	0.4636	0.8486	0.3820	0.4327
'rh.aparc_medialorbitofrontal '	0.7449	0.4627	0.3665	0.4743	0.6647	0.3941	0.8445
'rh.aparc_middletemporal '	0.6318	0.4142	0.4710	0.4823	0.5879	0.6097	0.8213
'rh.aparc_parahippocampal '	0.5788	0.7469	0.5920	0.4209	0.6231	0.0171	0.7498
'rh.aparc_paracentral '	0.9949	0.9393	0.4853	0.5910	0.5460	0.5243	0.5215
'rh.aparc_parsopercularis '	0.5543	0.3802	0.4504	0.4299	0.7840	0.8532	0.7443
'rh.aparc_parsorbitalis '	0.7170	0.3047	0.3909	0.3608	0.2005	0.3192	0.0587
'rh.aparc_parstriangularis '	0.4395	0.2914	0.4865	0.4515	0.6617	0.8020	0.1792
'rh.aparc_pericalcarine '	0.5189	0.3191	0.3522	0.0535	0.2558	0.3550	0.0078
'rh.aparc_postcentral '	0.5078	0.5262	0.5805	0.3204	0.0624	0.1664	0.1397
'rh.aparc_posteriorcingulate '	0.4781	0.4792	0.5094	0.5884	0.6106	0.3154	0.9968
'rh.aparc_precentral '	0.5561	0.6285	0.5891	0.3437	0.0431	0.0566	0.6294
'rh.aparc_precuneus '	0.8014	0.6194	0.3611	0.4325	0.6200	0.7673	0.4844
'rh.aparc_rostralanteriorcingulate'	0.9586	0.9871	0.3723	0.4049	0.6405	0.9977	0.8849
'rh.aparc_rostralmiddlefrontal '	0.5962	0.4565	0.4894	0.4175	0.2514	0.7241	0.1998
'rh.aparc_superiorfrontal '	0.9281	0.7940	0.5417	0.4444	0.4753	0.8595	0.7757
'rh.aparc_superiorparietal '	0.8182	0.7125	0.4529	0.3615	0.6205	0.8469	0.5100
'rh.aparc_superiortemporal '	0.5567	0.4397	0.4635	0.3279	0.2106	0.0778	0.3723
'rh.aparc_supramarginal '	0.7318	0.6198	0.4682	0.4774	0.8823	0.7872	0.7095
'rh.aparc_frontalpole '	0.3259	0.2743	0.5239	0.5592	0.5912	0.6142	0.5349
'rh.aparc_temporalpole '	0.7396	0.6431	0.5809	0.3276	0.5308	0.8974	0.4611
'rh.aparc_transversetemporal '	0.4042	0.4151	0.5502	0.5230	0.6801	0.8300	0.6655
'rh.aparc_insula '	0.5169	0.3853	0.4126	0.4943	0.8835	0.7070	0.9292

P_CORR

'lh.aparc_bankssts '	0.4877	0.4137	0.4559	0.5399	0.9860	0.9998	0.9524
'lh.aparc_caudalanteriorcingulate '	0.5773	0.5913	0.4453	0.4685	0.9550	0.9836	0.8078
'lh.aparc_caudalmiddlefrontal '	0.4607	0.4363	0.4945	0.4989	0.9974	0.9994	0.9346
'lh.aparc_cuneus '	0.6543	0.5743	0.3099	0.4963	0.9908	1.0000	0.9544
'lh.aparc_entorhinal '	0.2693	0.3865	0.5095	0.5619	0.9836	1.0000	0.7127
'lh.aparc_fusiform '	0.7043	0.6387	0.4523	0.5125	0.7339	0.9934	0.6163
'lh.aparc_inferiorparietal '	0.6623	0.6031	0.3771	0.4837	0.9994	0.9978	0.6619
'lh.aparc_inferiortemporal '	0.5543	0.5233	0.4551	0.4953	0.9986	0.7976	0.7554
'lh.aparc_isthmuscingulate '	0.5283	0.5659	0.4613	0.6177	0.9996	1.0000	0.9440
'lh.aparc_lateraloccipital '	0.5335	0.3349	0.2729	0.4599	0.9676	0.9922	0.6943
'lh.aparc_lateralorbitofrontal '	0.7433	0.7806	0.5081	0.5271	0.9970	0.9994	0.9054
'lh.aparc_lingual '	0.6527	0.4835	0.3285	0.4831	0.9688	0.8294	0.5219
'lh.aparc_medialorbitofrontal '	0.5905	0.4825	0.4917	0.5401	0.9724	0.9940	0.8906
'lh.aparc_middletemporal '	0.5483	0.5553	0.4711	0.4859	0.9972	1.0000	0.8412
'lh.aparc_parahippocampal '	0.5463	0.6679	0.5437	0.5353	0.9934	0.9988	0.9924
'lh.aparc_paracentral '	0.6579	0.6655	0.4739	0.5397	0.8702	0.9760	0.7816
'lh.aparc_parsopercularis '	0.7495	0.6971	0.4389	0.5157	0.9754	0.9994	0.9680
'lh.aparc_parsorbitalis '	0.7552	0.6697	0.4755	0.4805	0.9916	0.9956	0.9478
'lh.aparc_parstriangularis '	0.7023	0.6305	0.4495	0.4557	0.9932	0.9960	0.9410
'lh.aparc_pericalcarine '	0.7754	0.7782	0.4047	0.5301	0.9604	0.9736	0.9858
'lh.aparc_postcentral '	0.6537	0.6427	0.4727	0.4499	0.6753	0.8312	0.9486
'lh.aparc_posteriorcingulate '	0.5327	0.5309	0.4529	0.3887	0.9822	0.9274	0.9388
'lh.aparc_precentral '	0.5565	0.6091	0.5119	0.4523	0.9298	0.9494	0.9820
'lh.aparc_precuneus '	0.6919	0.6381	0.4077	0.5409	0.9942	0.9998	0.9748
'lh.aparc_rostralanteriorcingulate'	0.6323	0.5953	0.4403	0.3955	0.9784	0.9880	0.8322
'lh.aparc_rostralmiddlefrontal '	0.5113	0.4841	0.5007	0.5271	0.6521	0.9744	0.6577
'lh.aparc_superiorfrontal '	0.7095	0.6559	0.4843	0.4557	0.9844	0.9886	0.9220
'lh.aparc_superiorparietal '	0.5627	0.5721	0.4267	0.4359	0.9932	0.9964	0.9384
'lh.aparc_superiortemporal '	0.4931	0.5293	0.5175	0.4285	0.9984	1.0000	0.9044
'lh.aparc_supramarginal '	0.7167	0.7065	0.4579	0.4619	0.9714	0.9906	0.9938
'lh.aparc_frontalpole '	0.7321	0.5491	0.3927	0.3135	0.9256	0.9408	0.3975
'lh.aparc_temporalpole '	0.7009	0.7770	0.5893	0.4533	0.9610	0.9726	0.9284
'lh.aparc_transversetemporal '	0.3735	0.3153	0.4665	0.6549	0.9874	0.9998	0.9822
'lh.aparc_insula '	0.6287	0.5655	0.4139	0.5135	0.9998	0.9998	0.9888

'rh.aparc_bankssts '	0.5985	0.5725	0.4409	0.4579	0.9518	0.9908	0.9550
'rh.aparc_caudalanteriorcingulate '	0.6883	0.7109	0.5279	0.4367	0.9072	0.9544	0.7624
'rh.aparc_caudalmiddlefrontal '	0.4577	0.4707	0.4503	0.3983	0.9744	0.9836	0.9088
'rh.aparc_cuneus '	0.5327	0.5109	0.4129	0.5611	0.9970	0.9958	0.9598
'rh.aparc_entorhinal '	0.3597	0.5041	0.5261	0.4381	0.9664	0.9942	0.8074
'rh.aparc_fusiform '	0.6537	0.5731	0.4099	0.5349	0.9834	1.0000	0.9298
'rh.aparc_inferiorparietal '	0.4645	0.4525	0.4409	0.4729	0.9846	0.9988	0.9070
'rh.aparc_inferiortemporal '	0.6567	0.6519	0.4589	0.4901	0.9904	0.9832	0.8994
'rh.aparc_isthmuscingulate '	0.6487	0.5943	0.3729	0.6147	0.9272	0.9980	0.9740
'rh.aparc_lateraloccipital '	0.5099	0.3791	0.3523	0.5131	0.9604	0.9656	0.9038
'rh.aparc_lateralorbitofrontal '	0.7095	0.6063	0.4211	0.4933	0.9986	0.9882	0.8780
'rh.aparc_lingual '	0.6485	0.4391	0.2599	0.5041	0.9998	0.9564	0.9316
'rh.aparc_medialorbitofrontal '	0.6883	0.5785	0.3663	0.5107	0.9894	0.9626	0.9846
'rh.aparc_middletemporal '	0.6365	0.5463	0.4443	0.5151	0.9840	0.9970	0.9826
'rh.aparc_parahippocampal '	0.6079	0.7087	0.5105	0.4787	0.9864	0.6543	0.9886
'rh.aparc_paracentral '	0.7766	0.7812	0.4519	0.5695	0.9894	0.9922	0.9314
'rh.aparc_parsopercularis '	0.5943	0.5233	0.4323	0.4843	0.9998	1.0000	0.9762
'rh.aparc_parsorbitalis '	0.6735	0.4607	0.3865	0.4313	0.9416	0.9748	0.6493
'rh.aparc_parstriangularis '	0.5241	0.4505	0.4529	0.4957	0.9892	0.9968	0.8210
'rh.aparc_pericalcarine '	0.5709	0.4739	0.3535	0.0716	0.9558	0.9770	0.3155
'rh.aparc_postcentral '	0.5661	0.6079	0.5069	0.4001	0.8352	0.9456	0.7892
'rh.aparc_posteriorcingulate '	0.5487	0.5877	0.4675	0.5687	0.9972	0.9120	0.9908
'rh.aparc_precentral '	0.5951	0.6545	0.5093	0.4177	0.7720	0.8440	0.9642
'rh.aparc_precuneus '	0.7089	0.6509	0.3621	0.4865	0.9974	0.9998	0.9402
'rh.aparc_rostralanteriorcingulate'	0.7668	0.7960	0.3711	0.4653	0.9868	0.9998	0.9864
'rh.aparc_rostralmiddlefrontal '	0.6183	0.5737	0.4547	0.4767	0.9550	0.9956	0.8346
'rh.aparc_superiorfrontal '	0.7552	0.7241	0.4875	0.4921	0.9754	1.0000	0.9786
'rh.aparc_superiorparietal '	0.7157	0.6943	0.4337	0.4321	0.9860	0.9984	0.9452
'rh.aparc_superiortemporal '	0.5951	0.5653	0.4409	0.4061	0.9450	0.8840	0.9166
'rh.aparc_supramarginal '	0.6819	0.6513	0.4425	0.5129	0.9958	1.0000	0.9718
'rh.aparc_frontalpole '	0.4479	0.4335	0.4759	0.5549	0.9846	0.9932	0.9498
'rh.aparc_temporalpole '	0.6861	0.6623	0.5073	0.4055	0.9804	0.9990	0.9360
'rh.aparc_transversetemporal '	0.5015	0.5467	0.4917	0.5365	0.9898	0.9976	0.9672
'rh.aparc_insula '	0.5701	0.5267	0.4029	0.5219	0.9996	0.9950	0.9880

FREESURFER FINDINGS

VOLUME

	Sz T	Sz P	Sz P_CORR	DD T	DD P	DD P_CORR
'aseg_Left-Lateral-Ventricle '	0.073	0.942	0.949	0.623	0.537	0.844
'aseg_Left-Inf-Lat-Vent '	-0.276	0.784	0.953	-0.640	0.527	0.850
'aseg_Left-Cerebellum-White-Matter '	-0.444	0.660	0.914	0.001	1.000	0.957
'aseg_Left-Cerebellum-Cortex '	0.287	0.776	0.920	0.593	0.557	0.851
'aseg_Left-Thalamus-Proper '	0.214	0.832	0.931	0.542	0.592	0.865
'aseg_Left-Caudate '	0.946	0.351	0.734	1.296	0.204	0.616
'aseg_Left-Putamen '	0.437	0.665	0.892	0.757	0.454	0.804
'aseg_Left-Pallidum '	0.528	0.601	0.872	0.814	0.422	0.786
'aseg_3rd-Ventricle '	0.957	0.345	0.730	0.946	0.351	0.742
'aseg_4th-Ventricle '	0.338	0.738	0.912	-0.142	0.888	0.968
'aseg_Brain-Stem '	-0.061	0.951	0.966	0.236	0.815	0.926
'aseg_Left-Hippocampus '	1.080	0.288	0.692	1.509	0.141	0.527
'aseg_Left-Amygdala '	1.193	0.241	0.648	1.519	0.138	0.525
'aseg_CSF '	0.908	0.371	0.748	0.978	0.335	0.733
'aseg_Left-Accumbens-area '	0.109	0.914	0.945	-0.060	0.953	0.964
'aseg_Left-VentralDC '	0.148	0.883	0.940	0.378	0.708	0.901
'aseg_Left-vessel '	0.191	0.850	0.934	0.742	0.463	0.807
'aseg_Left-choroid-plexus '	1.326	0.194	0.596	1.970	0.057	0.330
'aseg_Right-Lateral-Ventricle '	0.400	0.691	0.899	0.760	0.453	0.803
'aseg_Right-Inf-Lat-Vent '	0.025	0.981	0.956	-0.114	0.910	0.970
'aseg_Right-Cerebellum-White-Matter'	-0.536	0.595	0.889	-0.148	0.883	0.968
'aseg_Right-Cerebellum-Cortex '	0.266	0.792	0.923	0.579	0.566	0.855
'aseg_Right-Thalamus-Proper '	0.372	0.712	0.905	0.691	0.495	0.822
'aseg_Right-Caudate '	0.814	0.422	0.786	1.002	0.324	0.724
'aseg_Right-Putamen '	0.245	0.808	0.927	0.571	0.572	0.858
'aseg_Right-Pallidum '	0.366	0.717	0.906	0.539	0.594	0.866
'aseg_Right-Hippocampus '	0.788	0.436	0.796	1.202	0.238	0.651
'aseg_Right-Amygdala '	0.878	0.386	0.761	1.246	0.222	0.634
'aseg_Right-Accumbens-area '	0.664	0.511	0.831	0.774	0.445	0.799
'aseg_Right-VentralDC '	0.054	0.957	0.950	0.306	0.761	0.916
'aseg_Right-vessel '	-0.227	0.822	0.961	0.437	0.665	0.889
'aseg_Right-choroid-plexus '	0.023	0.981	0.957	0.488	0.629	0.878
'aseg_5th-Ventricle '	0.610	0.546	0.848	0.653	0.518	0.834
'aseg_WM-hypointensities '	-0.510	0.613	0.896	-0.226	0.823	0.958
'aseg_Left-WM-hypointensities '	NaN	NaN	0.000	NaN	NaN	0.000
'aseg_Right-WM-hypointensities '	NaN	NaN	0.000	NaN	NaN	0.000
'aseg_non-WM-hypointensities '	-0.664	0.512	0.843	-0.324	0.748	0.941
'aseg_Left-non-WM-hypointensities '	NaN	NaN	0.000	NaN	NaN	0.000
'aseg_Right-non-WM-hypointensities '	NaN	NaN	0.000	NaN	NaN	0.000
'aseg_Optic-Chiasm '	0.460	0.648	0.886	0.658	0.515	0.832
'aseg_CC_Posterior '	0.651	0.519	0.833	0.778	0.442	0.798
'aseg_CC_Mid_Posterior '	0.381	0.705	0.904	0.634	0.531	0.840
'aseg_CC_Central '	0.289	0.774	0.920	0.446	0.658	0.887
'aseg_CC_Mid_Anterior '	0.830	0.413	0.781	1.325	0.194	0.603
'aseg_CC_Anterior '	0.242	0.810	0.927	0.680	0.501	0.826
'wmparc_wm-lh-bankssts '	0.127	0.900	0.944	0.032	0.975	0.953
'wmparc_wm-lh-caudalanteriorcingulate	0.307	0.761	0.917	0.783	0.439	0.795
'wmparc_wm-lh-caudalmiddlefrontal '	0.611	0.545	0.848	0.958	0.345	0.739
'wmparc_wm-lh-cuneus '	-0.283	0.779	0.951	0.265	0.793	0.921
'wmparc_wm-lh-entorhinal '	0.477	0.636	0.884	0.890	0.380	0.760
'wmparc_wm-lh-fusiform '	-0.378	0.708	0.932	-0.019	0.985	0.959
'wmparc_wm-lh-inferiorparietal '	0.082	0.935	0.948	0.218	0.829	0.929
'wmparc_wm-lh-inferiortemporal '	0.061	0.952	0.950	0.519	0.607	0.870
'wmparc_wm-lh-isthmuscingulate '	0.388	0.701	0.902	0.776	0.443	0.799
'wmparc_wm-lh-lateraloccipital '	0.399	0.693	0.899	0.805	0.427	0.789
'wmparc_wm-lh-lateralorbitofrontal '	-0.155	0.878	0.970	0.064	0.949	0.948
'wmparc_wm-lh-lingual '	0.010	0.992	0.959	0.300	0.766	0.916
'wmparc_wm-lh-medialorbitofrontal '	0.794	0.433	0.793	1.004	0.322	0.723
'wmparc_wm-lh-middletemporal '	0.710	0.482	0.817	0.926	0.361	0.750
'wmparc_wm-lh-parahippocampal	-0.222	0.826	0.961	0.140	0.890	0.939
'wmparc_wm-lh-paracentral '	-0.051	0.960	0.965	0.167	0.868	0.936
'wmparc_wm-lh-parsopercularis '	-0.362	0.720	0.937	-0.043	0.966	0.962
'wmparc_wm-lh-parsorbitalis '	-0.607	0.548	0.864	-0.351	0.728	0.936
'wmparc_wm-lh-parstriangularis '	0.221	0.826	0.930	0.344	0.733	0.906
'wmparc_wm-lh-pericalcarine '	-0.329	0.744	0.942	-0.018	0.986	0.959
'wmparc_wm-lh-postcentral '	-0.301	0.765	0.949	-0.084	0.934	0.967
'wmparc_wm-lh-posteriorcingulate '	0.666	0.510	0.830	1.032	0.310	0.715
'wmparc_wm-lh-precentral '	0.205	0.839	0.932	0.409	0.685	0.895
'wmparc_wm-lh-precuneus '	-0.221	0.826	0.961	0.237	0.814	0.926
'wmparc_wm-lh-rostralanteriorcingulate	-0.058	0.954	0.966	0.288	0.775	0.918
'wmparc_wm-lh-rostralmiddlefrontal '	0.443	0.660	0.891	0.798	0.430	0.791
'wmparc_wm-lh-superiorfrontal '	-0.233	0.817	0.960	0.069	0.946	0.947
'wmparc_wm-lh-superiorparietal '	0.215	0.831	0.931	0.525	0.603	0.869
'wmparc_wm-lh-superiortemporal '	0.203	0.840	0.933	0.743	0.463	0.807
'wmparc_wm-lh-supramarginal '	-0.378	0.708	0.932	0.095	0.925	0.944
'wmparc_wm-lh-frontalpole '	-0.612	0.545	0.863	-0.303	0.764	0.946
'wmparc_wm-lh-temporalpole '	0.517	0.609	0.875	0.539	0.593	0.866
'wmparc_wm-lh-transversetemporal	0.024	0.981	0.957	0.562	0.578	0.861
'wmparc_wm-lh-insula '	0.151	0.881	0.940	0.495	0.624	0.876
'wmparc_wm-rh-bankssts '	-0.018	0.986	0.961	0.199	0.843	0.932
'wmparc_wm-rh-caudalanteriorcingulate	0.188	0.852	0.935	0.409	0.685	0.895
'wmparc_wm-rh-caudalmiddlefrontal	0.561	0.578	0.862	1.161	0.254	0.665
'wmparc_wm-rh-cuneus '	0.387	0.702	0.902	0.760	0.453	0.803
'wmparc_wm-rh-entorhinal '	0.217	0.830	0.931	0.866	0.392	0.768
'wmparc_wm-rh-fusiform '	-0.303	0.764	0.948	0.056	0.956	0.949
'wmparc_wm-rh-inferiorparietal '	0.522	0.605	0.874	0.854	0.399	0.772
'wmparc_wm-rh-inferiortemporal '	0.495	0.624	0.881	0.837	0.409	0.778
'wmparc_wm-rh-isthmuscingulate '	-0.032	0.975	0.963	0.444	0.660	0.887
'wmparc_wm-rh-lateraloccipital '	0.273	0.787	0.922	0.669	0.508	0.829
'wmparc_wm-rh-lateralorbitofrontal '	0.100	0.921	0.947	0.287	0.776	0.918
'wmparc_wm-rh-lingual '	0.239	0.813	0.928	0.603	0.551	0.850
'wmparc_wm-rh-medialorbitofrontal '	0.285	0.778	0.920	0.657	0.516	0.833
'wmparc_wm-rh-middletemporal '	0.005	0.996	0.959	0.391	0.699	0.899
'wmparc_wm-rh-parahippocampal	-0.237	0.814	0.959	0.148	0.883	0.938
'wmparc_wm-rh-paracentral '	0.009	0.993	0.959	0.022	0.982	0.954
'wmparc_wm-rh-parsopercularis '	0.143	0.887	0.941	0.459	0.649	0.884
'wmparc_wm-rh-parsorbitalis '	0.194	0.847	0.934	0.464	0.646	0.884
'wmparc_wm-rh-parstriangularis '	0.445	0.659	0.891	0.742	0.463	0.807
'wmparc_wm-rh-pericalcarine '	0.300	0.766	0.918	0.833	0.411	0.780
'wmparc_wm-rh-postcentral '	0.094	0.926	0.947	0.324	0.748	0.910
'wmparc_wm-rh-posteriorcingulate '	0.361	0.720	0.907	0.768	0.448	0.800
'wmparc_wm-rh-precentral '	0.074	0.941	0.949	0.281	0.781	0.918
'wmparc_wm-rh-precuneus '	-0.226	0.823	0.961	0.053	0.958	0.949
'wmparc_wm-rh-rostralanteriorcingulate	0.080	0.937	0.948	0.492	0.626	0.877
'wmparc_wm-rh-rostralmiddlefrontal '	0.101	0.920	0.947	0.534	0.597	0.867
'wmparc_wm-rh-superiorfrontal '	-0.298	0.768	0.949	0.006	0.995	0.956
'wmparc_wm-rh-superiorparietal '	-0.187	0.853	0.967	0.079	0.938	0.946
'wmparc_wm-rh-superiortemporal '	-0.211	0.835	0.963	0.182	0.857	0.934
'wmparc_wm-rh-supramarginal '	-0.121	0.905	0.971	0.330	0.744	0.909
'wmparc_wm-rh-frontalpole '	0.475	0.638	0.884	0.880	0.385	0.765
'wmparc_wm-rh-temporalpole '	0.470	0.641	0.885	0.800	0.429	0.791
'wmparc_wm-rh-transversetemporal	-0.136	0.893	0.973	0.333	0.741	0.908
'wmparc_wm-rh-insula '	0.294	0.770	0.919	0.706	0.485	0.817
'wmparc_Left-UnsegmentedWhiteMatte	0.106	0.917	0.946	0.638	0.528	0.839
'wmparc_Right-UnsegmentedWhiteMat	0.096	0.924	0.947	0.602	0.551	0.850

GROSS STATS	Sz T	Sz P	DD T	DD P
'lhCortexVol'	0.381	0.705	0.742	0.463
'rhCortexVol'	0.386	0.702	0.721	0.476
'CortexVol'	0.384	0.704	0.732	0.469
'lhCorticalWhiteMatterVol'	0.070	0.945	0.434	0.667
'rhCorticalWhiteMatterVol'	0.077	0.939	0.448	0.657
'CorticalWhiteMatterVol'	0.073	0.942	0.442	0.662
'SubCortGrayVol'	0.517	0.609	0.833	0.411
'TotalGrayVol'	0.378	0.708	0.716	0.479
'SupraTentorialVol'	0.252	0.803	0.617	0.541
'SupraTentorialVolNotVent'	0.250	0.804	0.609	0.547
'SupraTentorialVolNotVentVox'	0.249	0.805	0.607	0.548
eITV	0.392	0.697	0.722	0.475

GROSS PROPORTIONS				
'lh_LR_length'	0.604	0.550	0.838	0.408
'lh_PA_length'	0.316	0.754	0.435	0.666
'lh_IS_length'	0.600	0.553	0.676	0.504
'lh_LR_PA_ratio'	0.907	0.371	1.044	0.304
'lh_LR_IS_ratio'	0.618	0.541	0.800	0.429
'lh_PA_IS_ratio'	0.353	0.727	0.424	0.674
'rh_LR_length'	0.695	0.492	0.968	0.340
'rh_PA_length'	0.324	0.748	0.451	0.655
'rh_IS_length'	0.509	0.614	0.651	0.520
'rh_LR_PA_ratio'	0.984	0.332	1.154	0.257
'rh_LR_IS_ratio'	0.784	0.439	0.936	0.356
'rh_PA_IS_ratio'	0.447	0.658	0.458	0.650
'LR_asymmetry'	0.562	0.578	0.552	0.585
'PA_asymmetry'	0.615	0.543	0.630	0.533
'IS_asymmetry'	0.716	0.479	0.666	0.510

PCA RESULTS

Component rank	Sz			DD			BIN		
	T	P	P_CORR	T	P	P_CORR	T	P	P_CORR
1.000	-2.657	0.012	0.055	-1.746	0.091	0.818	-1.080	0.288	0.998
2.000	1.307	0.201	0.996	1.273	0.212	0.997	0.512	0.612	1.000
3.000	0.931	0.359	1.000	0.956	0.346	1.000	0.477	0.637	1.000
4.000	-0.675	0.504	1.000	-1.671	0.105	0.866	-1.239	0.225	0.991
5.000	0.233	0.817	1.000	-0.236	0.815	1.000	-0.640	0.527	1.000
6.000	-0.015	0.988	1.000	0.025	0.981	1.000	0.194	0.848	1.000
7.000	-1.302	0.202	0.984	-1.775	0.086	0.798	0.240	0.812	1.000
8.000	-2.560	0.016	0.055	-3.033	0.005	0.027	-2.554	0.016	0.199
9.000	-0.349	0.729	1.000	0.020	0.984	1.000	-0.971	0.339	1.000
10.000	0.992	0.329	1.000	0.373	0.712	1.000	0.703	0.487	1.000
11.000	-1.408	0.169	0.964	-1.607	0.118	0.900	-2.149	0.040	0.500
12.000	0.856	0.398	1.000	0.629	0.534	1.000	-0.361	0.720	1.000
13.000	-1.401	0.171	0.966	-0.792	0.434	1.000	0.177	0.861	1.000
14.000	0.299	0.767	1.000	0.537	0.595	1.000	0.435	0.666	1.000
15.000	0.417	0.680	1.000	0.398	0.694	1.000	0.771	0.446	1.000
16.000	0.104	0.918	1.000	0.703	0.487	1.000	-0.287	0.776	1.000
17.000	-0.790	0.435	1.000	-0.963	0.343	0.999	1.227	0.229	0.994
18.000	1.168	0.252	0.999	1.274	0.212	0.997	1.827	0.077	0.829
19.000	-0.253	0.802	1.000	-1.062	0.297	0.997	-0.451	0.655	1.000
20.000	-1.235	0.226	0.990	-0.979	0.335	0.999	-2.127	0.042	0.522
21.000	-1.580	0.124	0.903	-0.703	0.487	1.000	-0.821	0.418	1.000
22.000	-0.380	0.707	1.000	-1.145	0.261	0.995	-0.376	0.710	1.000
23.000	1.044	0.305	1.000	1.038	0.307	1.000	0.640	0.527	1.000
24.000	-0.442	0.662	1.000	0.151	0.881	1.000	0.550	0.586	1.000
25.000	-1.064	0.295	0.998	-1.158	0.256	0.994	-1.468	0.152	0.959
26.000	-1.339	0.190	0.979	-1.280	0.210	0.987	-0.963	0.343	1.000
27.000	-0.686	0.498	1.000	-0.371	0.713	1.000	0.365	0.718	1.000
28.000	0.259	0.797	1.000	0.109	0.914	1.000	0.525	0.603	1.000
29.000	0.642	0.525	1.000	0.508	0.615	1.000	-1.295	0.205	0.987
30.000	-0.324	0.748	1.000	-0.872	0.390	1.000	-1.242	0.224	0.991
31.000	-0.869	0.391	1.000	-1.053	0.300	0.997	0.230	0.819	1.000
32.000	1.196	0.241	0.999	0.024	0.981	1.000	-0.170	0.866	1.000
33.000	0.305	0.762	1.000	0.552	0.585	1.000	0.829	0.413	1.000
34.000	-0.392	0.698	1.000	-0.083	0.934	1.000	-0.484	0.632	1.000
35.000	-0.016	0.987	1.000	0.196	0.846	1.000	0.694	0.493	1.000

PCA weights for variables associated with component 8

Ordered by weight (first	Direction	Weight
'WMmicro_vol_CCbody	1.000	0.594
'WMmicro_vol_CCsplen	-1.000	0.502
'WHOLEBRAIN_BrainS	1.000	0.192
'WMmicro_vol_FORNIX	-1.000	0.162
'WMmicro_vol_FORNIX	-1.000	0.162
'WMmicro_vol_corticoS	1.000	0.156
'WMmicro_vol_corticoS	1.000	0.149
'WMmicro_vol_ARCL'	-1.000	0.146
'WMmicro_vol_Cing_R'	1.000	0.131
'WHOLEBRAIN_Supra	-1.000	0.126
'WHOLEBRAIN_Supra	-1.000	0.113
'WMmicro_vol_IFOL'	-1.000	0.110
'WMmicro_vol_CCgenu	1.000	0.104
'GM_vol_aseg_Right-La	1.000	0.102
'WMmicro_vol_Cing_L'	1.000	0.101
'WMmicro_vol_ilfLeft'	-1.000	0.100
'WHOLEBRAIN_lhCorti	1.000	0.091
'WMmicro_vol_UNCR'	1.000	0.084
'WHOLEBRAIN_SubCo	-1.000	0.082
'GM_thick_avg_lh.apar	-1.000	0.076
'GM_thick_avg_rh.apar	-1.000	0.074
'WMmicro_vol_PHCR'	-1.000	0.073
'GM_thick_avg_lh.apar	-1.000	0.066
'GM_thick_avg_rh.apar	-1.000	0.064
'WHOLEBRAIN_Cortex	-1.000	0.064
'GM_vol_aseg_Right-C	1.000	0.062
'GM_thick_avg_lh.apar	1.000	0.058
'GM_thick_avg_lh.apar	-1.000	0.058
'GM_vol_wmparc_Righ	-1.000	0.056
'GM_vol_wmparc_Left-	-1.000	0.053
'WMmicro_vol_ARCR'	-1.000	0.053
'WHOLEBRAIN_rhCort	-1.000	0.052
'GM_vol_aseg_Left-Ce	1.000	0.048
'GM_vol_wmparc_wm-l	1.000	0.047
'GM_vol_wmparc_wm-l	-1.000	0.047
'WHOLEBRAIN_rhCort	-1.000	0.047
'GM_vol_aseg_Brain-S	-1.000	0.047
'GM_thick_avg_rh.apar	1.000	0.046
'GM_thick_avg_lh.apar	-1.000	0.045
'GM_thick_avg_lh.apar	1.000	0.042
'GM_vol_wmparc_wm-r	1.000	0.038
'WHOLEBRAIN_Cortica	1.000	0.038
'GM_surf_area_lh.apar	-1.000	0.038
'GM_thick_avg_lh.apar	1.000	0.037
'WMmicro_vol_PHCL'	-1.000	0.033
'WMmicro_vol_ilfRight'	1.000	0.032
'GM_thick_avg_lh.apar	-1.000	0.032
'GM_vol_wmparc_wm-l	-1.000	0.031
'GM_thick_avg_lh.apar	1.000	0.031
'GM_vol_wmparc_wm-l	-1.000	0.029

POST-HOC TEST OF RATIO CCBODY-CCSPLENIUM

	T	P
Sz	-2.180	0.037
DD	-2.979	0.006

CHANGE IN SIZE IN IMPLICATED WM REGIONS

JHU reegion	JHU index	change	
Ccbody	4	0.594	
Ccsplenium	5	-0.502	
		0.192	
FornixL	6	0.149	
FornixR	6	-0.146	
CorticoSpinalL	8	-0.162	
CorticoSpinalR	7	0.131	
ArcuateL	42	0.156	JHU dosnt have Arcuate. Using SLF as approximation
		-0.126	
		-0.113	
CingulumR	35	-0.110	
Ccgenu	3	-0.162	
		0.102	
UncinateR	45	0.101	
CingulumL	36		
ParahippCingR	37		
